# Co-opted Oxysterol-Binding ORP and VAP Proteins Channel Sterols to RNA Virus Replication Sites via Membrane Contact Sites

**DOI:** 10.1371/journal.ppat.1004388

**Published:** 2014-10-16

**Authors:** Daniel Barajas, Kai Xu, Isabel Fernández de Castro Martín, Zsuzsanna Sasvari, Federica Brandizzi, Cristina Risco, Peter D. Nagy

**Affiliations:** 1 Department of Plant Pathology, University of Kentucky, Lexington, Kentucky, United States of America; 2 Cell Structure Laboratory, Centro Nacional de Biotecnología (CNB-CSIC), Campus de Cantoblanco, Madrid, Spain; 3 MSU-DOE Plant Research Laboratory, Michigan State University, East Lansing, Michigan, United States of America; Agriculture and Agri-Food Canada, Canada

## Abstract

Viruses recruit cellular membranes and subvert cellular proteins involved in lipid biosynthesis to build viral replicase complexes and replication organelles. Among the lipids, sterols are important components of membranes, affecting the shape and curvature of membranes. In this paper, the tombusvirus replication protein is shown to co-opt cellular Oxysterol-binding protein related proteins (ORPs), whose deletion in yeast model host leads to decreased tombusvirus replication. In addition, tombusviruses also subvert Scs2p VAP protein to facilitate the formation of membrane contact sites (MCSs), where membranes are juxtaposed, likely channeling lipids to the replication sites. In all, these events result in redistribution and enrichment of sterols at the sites of viral replication in yeast and plant cells. Using *in vitro* viral replication assay with artificial vesicles, we show stimulation of tombusvirus replication by sterols. Thus, co-opting cellular ORP and VAP proteins to form MCSs serves the virus need to generate abundant sterol-rich membrane surfaces for tombusvirus replication.

**Authors Summary**

Cellular proteins and cellular membranes are usurped by positive-stranded RNA viruses to assemble viral replicase complexes required for their replication. Tombusviruses, which are small RNA viruses of plants, depend on sterol-rich membranes for replication. The authors show that the tombusviral replication protein binds to cellular oxysterol-binding ORP proteins. Moreover, the endoplasmic reticulum resident cellular VAP proteins also co-localize with viral replication proteins. These protein interactions likely facilitate the formation of membrane contact sites that are visible in cells replicating tombusvirus RNA. The authors also show that sterols are recruited and enriched to the sites of viral replication. In vitro replication assay was used to show that sterols indeed stimulate tombusvirus replication. In summary, tombusviruses use subverted cellular proteins to build sterol-rich membrane microdomain to promote the assembly of the viral replicase complex. The paper connects efficient virus replication with cellular lipid transport and membrane structures.

## Introduction

Plus-stranded (+)RNA viruses subvert various intracellular and organellar membranes to assemble viral replicase complexes (VRCs) consisting of viral replication proteins and co-opted host proteins and the viral RNAs in the infected cells [1]–[6]. Although several of the co-opted host proteins affect the biochemical activities of the viral RNA-dependent RNA polymerases (RdRp), [7]–[11], other host proteins, such as the ESCRT proteins, reticulons and amphiphysins are proposed to function by facilitating membrane deformation occurring during VRC assembly [12]–[15], further highlighting the importance of membranes in viral replication.

RNA viruses also subvert cellular proteins involved in lipid biosynthesis or alter intracellular lipid metabolism and lipid transport [5], [6], [16], [17]. Viral RdRps of many (+)RNA viruses interact with membranes and build functional VRCs in single-membrane spherules and vesicle-like structures that have a narrow opening to the cytosol. Accordingly, virus-induced formation of spherules, double membrane vesicles or tubulovesicular cubic membranes is documented in a variety of cell organelles [6], [16], [18].

The virus-induced VRCs and membranous structures not only gather all the replication factors into confined cytosolic areas, but importantly, they also protect the fragile viral RNAs from degradation by host ribonucleases and help avoid recognition of viral components by the host antiviral surveillance system [6], [19]. Overall, assembly of the VRCs is an essential step during the replication of (+)RNA viruses that is absolutely dependent on cellular lipids and membranes in the infected cells.

*Tomato bushy stunt virus* (TBSV) and *Carnation Italian ringspot virus* (CIRV) are tombusviruses with small (+)RNA genome that serve as models to study virus - host interactions, virus replication and recombination using yeast (*Saccharomyces cerevisiae*) as a surrogate host [6], [20]–[22]. Tombusviruses code for two replication proteins involved in all the replication steps. The auxiliary TBSV p33 replication protein, which is an RNA chaperone, recruits the TBSV (+)RNA to the site of replication, which occurs at the cytosolic surface of peroxisomal membranes, while the auxiliary CIRV p36 replication protein recruits the viral (+)RNA to the outer mitochondrial membrane [23]–[25]. The interaction between the TBSV RdRp protein p92^pol^ and the p33 replication protein is required for assembling the functional VRCs [26]. Multiple systematic genome-wide screens in combination with global proteomics approaches have led to the identification of ∼500 host proteins/genes that are implicated in TBSV replication and recombination [7], [9], [27]–[35]. The tombusvirus p33 and p92^pol^ replication proteins, the viral (+)RNA and a ∼dozen co-opted host proteins are recruited to peroxisome membranes to assemble the tombusvirus VRCs [6], [15], [26], [34]–[45].

Previously, we have shown that TBSV replication depends on sterol and phospholipid biosynthesis in yeast and plant cells [46], [47]. Sterols are ubiquitous and essential membrane components in all eukaryotes, regulating many membrane functions, membrane rigidity, fluidity, curvature and permeability by interacting with other lipids and proteins within the membranes [48]–[50]. Sterols are also important for the organization of detergent-resistant membrane microdomains, called membrane rafts [51]. Most sterols are synthesized in the ER membrane and mainly accumulate in the plasma membrane in uninfected cells.

The lipid transfer proteins, including the sterol- and oxysterol-binding proteins [OSBP or Oxysterol-binding protein related proteins (ORPs)] are involved in many cellular processes, which includes nonvesicular sterol transfer, vesicular trafficking, lipid metabolism, and signaling [52]–[55]. The ORPs are important to increase the local concentration of sterols in membrane compartments that facilitate the bending of membranes [52]–[55]. Yeast has seven ORP genes (*OSH1-7*), while *Arabidopsis* has 12 predicted ORPs [56]. Because of these features, ORPs might facilitate the formation of viral replication organelles, including VRCs when subverted by viruses.

The ORPs are known to function at membrane contact sites (MCS or ER junctions), where the ER membrane is proximal to other intracellular organelles [57], [58]. MCSs are proposed to facilitate the non-vesicular trafficking of small molecules, including sterols and other lipids. The ORPs are recruited to MCSs by VAP (VAMP-associated protein) proteins [57], [58]. VAPs are present in all eukaryotes and implicated in the regulation of lipid metabolism and transport, membrane trafficking, microtubule organization and the unfolded protein response [59]. Interestingly, the global proteomics screens with TBSV identified the p33-interacting Scs2p protein [33], which is the major member of the VAP family in yeast. Arabidopsis has 10 VAP orthologs, which are grouped in the VAP33 subfamily and the best-characterized member, PVA12 is known to localize to the ER, similar to the yeast Scs2p VAP protein [56]. Scs2p is a tail-anchored, ER membrane-associated protein that anchors the ORPs to the ER membrane [58].

In this paper, we identify the yeast oxysterol-binding homology (Osh) proteins, which are co-opted to support tombusvirus replication. We also show the redistribution and enrichment of sterols to the site of tombusvirus replication, suggesting that the viral replication proteins interact with the yeast Osh proteins to create sterol-rich membrane microdomains. In addition, we present evidence that the viral replication proteins bind to the yeast Scs2p VAP protein in the ER membrane, likely facilitating the formation of MCSs and the recruitment of Osh proteins. Many of our findings are also observed in plants, suggesting that the subversion of ORPs and VAP proteins by tombusviruses also takes place in plants. Altogether, these virus-host interaction processes likely serve the virus need to generate abundant sterol-rich membrane surfaces for virus replication.

## Results

### Tombusvirus replication depends on recruited cellular oxysterol-binding proteins in yeast model host

**Figure 1:**
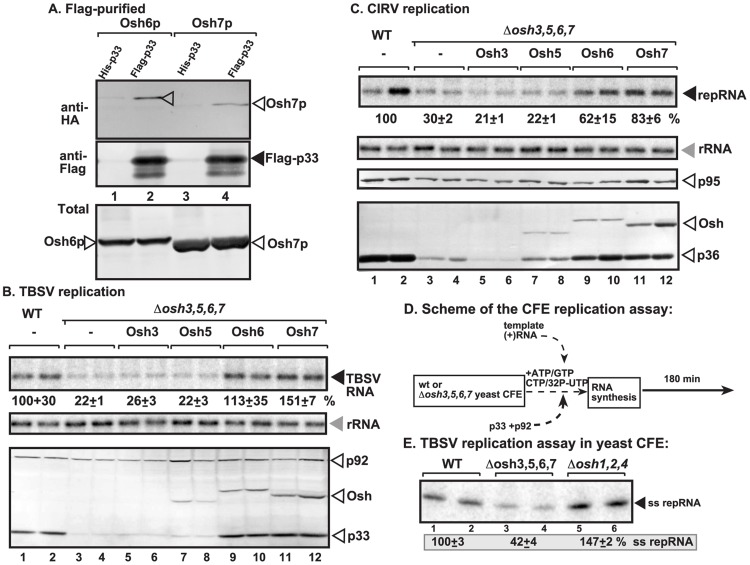
Interaction between p33 replication protein and yeast oxysterol-binding Osh proteins. (A) Co-purification of the yeast Osh6p and Osh7p proteins with the p33 replication protein. Top panel: Western blot analysis of co-purified HA-tagged cellular proteins with Flag-affinity purified p33 from isolated membrane fraction of yeast cells. Osh6p and Osh7p were detected with anti-HA antibody. The negative control was His_6_-tagged p33 purified from yeast extracts using a FLAG-affinity column. Middle panel: Western blot of purified Flag-p33 detected with anti-FLAG antibody. Bottom panel: Western blot of HA-tagged Osh6p and Osh7p proteins in the total yeast extracts using anti-HA antibody. (B) Decreased TBSV repRNA accumulation in *osh3,5,6,7Δ* yeast. To launch TBSV repRNA replication, we expressed His_6_-p33 from the galactose-inducible *GAL1* promoter, His_6_-p92 from the copper-inducible *CUP1* promoter and DI-72(+) repRNA from the galactose-inducible *GAL10* promoter in the parental (SEY6210) and in *osh3,5,6,7Δ* yeast strains. His_6_-tagged Osh3, 5, 6, 7 were expressed from *GAL1* promoter. The yeast cells were pre-cultured for 24 hours at 23°C in 2% glucose SC minimal media, and then for 48 h at 23°C in 2% galactose SC minimal media supplemented with 50 µM CuSO_4_. Northern blot analysis was used to detect DI-72(+) repRNA accumulation. The accumulation level of DI-72(+) repRNA was normalized based on 18S rRNA levels. Bottom panel: Western blot analysis of the accumulation level of His_6_-tagged p33, His_6_-p92 and His_6_-Osh proteins using anti-His antibodies. Each experiment was performed three times. (C) Decreased accumulation of the mitochondrial CIRV in *osh3,5,6,7Δ* yeast. See further details in Panel B. (D) Scheme of the *in vitro* tombusvirus replicase assay based on yeast CFEs and purified recombinant TBSV replication proteins. (E) Reduced activity of the tombusvirus replicase assembled in CFE from *osh3,5,6,7Δ* yeast. Denaturing PAGE analysis of *in vitro* tombusvirus replicase activity in the CFEs. Note that this image shows the repRNAs made by a full cycle of replicase activity, producing both (−) and (+)-strands, *in vitro*. The CFEs contained the same amount of total yeast proteins (not shown). Each experiment was performed three times.

Tombusvirus replication greatly depends on sterols [47], which are distributed in cells via vesicle transfer and sterol-binding ORP proteins in a vesicle independent pathway [53], [55]. Therefore, we have tested if TBSV replication proteins interact with the yeast ORPs, named Osh1-7p, to facilitate sterol transfer in virus-infected cells. Our co-purification experiments with the affinity-purified tombusvirus p33 replication protein from isolated membranous fraction of yeast model host showed that four of the seven yeast Osh proteins, which are lipid-transfer proteins involved in oxysterol/sterol-binding, were associated with the membrane-bound p33 (Fig. 1A and S1A). These yeast proteins, namely Osh3p, Osh5p, Osh6p and Osh7p, were not only efficiently co-purified with p33 replication protein, but they also bound directly to p33 in a pull-down assay (Fig. S1B) and interacted with p33 in a membrane (split-ubiquitin-based) yeast two-hybrid assay (Fig. S1C). Co-purification of Osh4p was less robust with p33 from membranous fraction of yeast, while the co-purified Osh1p and Osh2p were close to the detection limit (Fig. S1A).

To demonstrate that some members of the 12 ORP proteins from *Arabidopsis thaliana* plants [60] could also interact with the tombusvirus p33 replication protein, we expressed 6 small ORP (i.e., ORP3A-C and ORP4A-C) proteins from *A. thaliana*, which similar to the yeast Osh6p lack the FFAT motif, in yeast cells replicating TBSV repRNA. The FFAT motif in mammalian ORPs and several of the yeast ORPs is used for interaction with VAPs [53]. Affinity-purification of tombusvirus p33 from isolated membranous fraction of yeast co-expressing one of the AtORPs showed that all six AtORP proteins were co-purified with p33 (Fig. S2). Altogether, these data have established that a set of host ORPs directly interacted with the tombusvirus p33 replication protein and these cytosolic proteins are likely recruited to intracellular membranes by p33 replication protein.

To study if the recruited cellular Osh proteins affect TBSV replication in yeast, we used a yeast strain lacking the four p33-interacting *OSH* genes (*osh3,5,6,7Δ*) [61]. Interestingly, replication of TBSV RNA decreased by ∼5-fold in *osh3,5,6,7Δ* yeast in comparison with the wt yeast (compare lanes 3–4 with 1–2 in Fig. 1B). The amount of the p33 replication protein accumulating in *osh3,5,6,7Δ* yeast greatly decreased, suggesting that these cellular Osh proteins are needed for the stability of this viral replication protein, while their lesser effects on p92^pol^ replication protein accumulation was also observed (Fig. 1B, lanes 3–4; also, Fig. S1D, lanes 3–4). Similarly, another tombusvirus, CIRV, which unlike TBSV that utilizes the peroxisome membrane, takes advantage of the mitochondrial outer membranes for replication [23]–[25], also accumulated at a ∼3-fold reduced level in *osh3,5,6,7Δ* yeast (compare lanes 3–4 with 1–2 in Fig. 1C). The accumulation of the CIRV p36 and p95^pol^ replication proteins also decreased in *osh3,5,6,7Δ* yeast. Based on our findings that single deletion of each of the seven yeast *OSH* genes (not shown) or combined deletion of three genes in *osh5,6,7Δ* or *osh1,2,4Δ* yeasts (Fig. S1D) did not have a major effect on TBSV replication, while combined deletion of the four *OSH* genes in *osh3,5,6,7Δ* yeast decreased TBSV and CIRV accumulation, we suggest that Osh3/5/6/7 proteins likely play at least partially overlapping roles in tombusvirus replication.

Complementation studies with individually expressed Osh proteins in *osh3,5,6,7Δ* yeast revealed that Osh6p and Osh7p could fully complement TBSV replication, while also partially complementing CIRV replication (lanes 9–12, Fig. 1B–C). Complementation of tombusvirus replication by Osh3p and Osh5p was not detectable under these conditions (lanes 5–8, Fig. 1B–C).

To obtain evidence that Osh proteins can affect the assembly of the membrane-bound tombusvirus replicase complex, we took advantage of a cell-free TBSV replication assay based on yeast cell-free extracts (CFE) [26]. In this assay, the TBSV replicase is assembled in the test tube using CFE, purified recombinant p33 and p92^pol^ and *in vitro* transcribed TBSV (+)repRNA (Fig. 1D) [26]. Under these experimental conditions, the CFE can support a single round of full tombusviral replication, producing ^32^P-labeled minus- (which is present in dsRNA form) and excess amount of viral plus-strand RNA progeny [26], [62]. We found that CFE prepared from *osh3,5,6,7Δ* yeast supported TBSV RNA replication only with ∼40% efficiency when compared with CFE from wt yeast (compare lanes 3–4 with 1–2 in Fig. 1E). In contrast, CFE prepared from *osh1,2,4Δ* yeast lacking the three Osh proteins not interacting with p33 (Fig. 1E), supported *in vitro* TBSV replication efficiently, suggesting that, unlike Osh3/5/6/7; the Osh1p, Osh2p and Osh4p ORP proteins are not required for the *in vitro* assembly of the TBSV replicase.

Overall, these data indicate that Osh3p, Osh5p, Osh6p and Osh7p are required for TBSV and CIRV RNA replication, as well as that they affect replicase assembly and the accumulation of tombusvirus replication proteins. However, these proteins likely play redundant roles since combined deletion of the four p33-interacting *OSH* genes was needed to have substantial effect on TBSV accumulation in yeast and yet, single expression of either Osh6p or Osh7p in *osh3,5,6,7Δ* yeast was satisfactory to complement the viral replication defect in *osh3,5,6,7Δ* yeast.

### Co-localization of Osh6p with tombusvirus replication proteins in yeast

**Figure 2:**
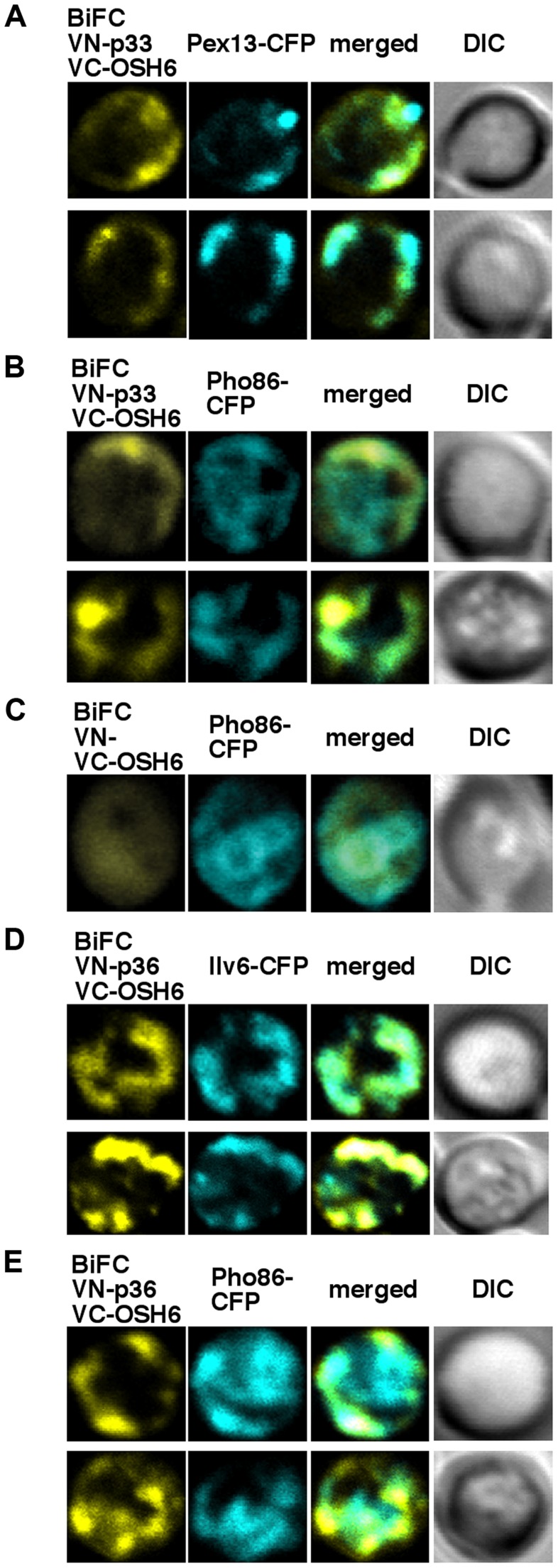
The tombusvirus p33 replication protein interacts with the yeast Osh6p protein in the ER. (A–B) Left panels: BiFC analysis of interaction between Osh6p and p33 in yeast cells. Confocal laser microscopy images also show the peroxisomal localization of Pex13-CFP or ER-localized Pho86-CFP. The merged images demonstrate the interaction between p33 and Osh6p (detected via BiFC) and their partial co-localization with Pex13-CFP and Pho86-CFP. DIC (differential interference contrast) images are shown on the right. (C) Control BiFC experiments with yeast lacking the p33 sequence. Yeast was grown under similar conditions and images were taken as in panel A–B. (D–E) BiFC analysis of interaction between Osh6p and the CIRV p36 in yeast cells. Confocal laser microscopy images show the mitochondrial localization of Ilv6-CFP or ER-localized Pho86-CFP. The merged images demonstrate the interaction between p36 and Osh6p and their partial co-localization with Ilv6-CFP and Pho86-CFP.

To test if Osh6p interacts and co-localizes with the tombusvirus p33 replication protein, we used bimolecular fluorescence complementation (BiFC) assay in yeast co-expressing the N-terminal half of Venus YFP fused to p33 and the C-terminal half of Venus YFP fused to Osh6p (Fig. 2A). The BiFC signal revealed interaction between p33 and Osh6p and these proteins were co-localized partially to the peroxisome membranes (Fig. 2A) and to the ER membrane (Fig. 2B). The BiFC signal with Osh6p in the absence of the p33 fusion part (i.e., expression of the N-terminal half of Venus YFP only) was close to background level (Fig. 2C) [63]. Using the BiFC approach, we also observed interaction between the CIRV p36 replication protein and Osh6p and partial co-localization to the mitochondrial membrane (Fig. 2D) and to the ER membrane (Fig. 2E). Thus, the mitochondria-localized CIRV p36 could also co-opt Osh6p, but the relocalization of Osh6p occurs at a different subcellular location than in the case of peroxisome-localized TBSV. Overall, these data support that Osh6p is recruited by tombusviruses to subcellular membranes likely via interaction between the replication proteins and Osh6p.

### Re-distribution of sterols to the sites of tombusvirus replication in yeast

**Figure 3:**
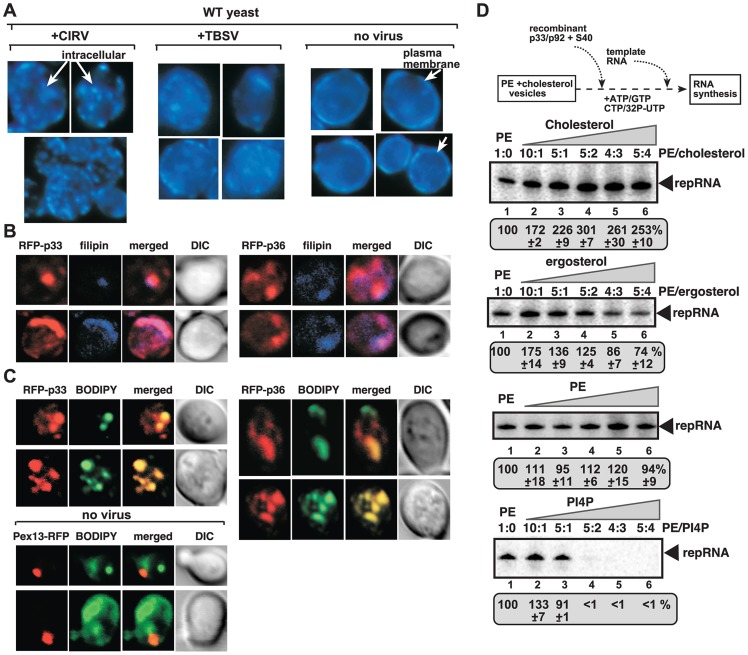
Enrichment of sterols at the sites of tombusvirus replication in yeast. (A) Re-localization of ergosterols to internal punctate structures in yeast replicating CIRV or TBSV. Fluorescent microscopic images of yeast cells stained with filipin dye. Note that filipin stains ergosterols present mostly at the plasma membrane in virus-free cells. (B) Co-localization of ergosterols and CIRV RFP-p36 and CNV RFP-p33 is shown by confocal laser microscopy. Yeasts were stained with filipin dye. (C) Enrichment of fluorescently-labeled sterol at the sites of tombusvirus replication in yeast deficient in ergosterol synthesis (*erg9Δ*). The BODIPY-cholesterol was taken up by yeast from the culture media. The bottom two rows represent images from yeast not expressing tombusvirus proteins and serve as control. (D) Sterols stimulate *in vitro* TBSV replication in artificial vesicles. Artificial PE vesicles (liposomes) were made in the presence of increasing concentrations of cholesterol or ergosterol, followed by *in vitro* TBSV replication assay as shown schematically. The amount of TBSV repRNA synthesized in the *in vitro* assay is shown in PAGE images. PE and PI(4)P were used as controls. Note that addition of extra PE did not change TBSV replication (second panel from the bottom), while PI(4)P inhibited it when used at higher concentrations (bottom image).

Since the proposed major function of ORPs is to transfer sterols between organellar membranes inside cells [52], [64], we predicted that recruitment of Osh3/5/6/7 ORPs by tombusviruses to the sites of viral replication could result in enrichment of sterols in these viral subcompartments. Accordingly, testing the distribution of sterols in yeast using the fluorescent sterol probe filipin dye [61] revealed striking differences between yeast cells replicating or not replicating tombusviruses. Namely, the sterols, which are mostly enriched in the plasma membrane in yeast reaching ∼60% of total ergosterols (images without tombusviruses on the right, Fig. 3A) [53], were redistributed to internal punctate-like subcellular compartments in yeast replicating TBSV and CIRV tombusviruses (left and central images). The redistributed sterols were mostly present in punctate-like structures in yeast replicating the mitochondria-localized CIRV, while the punctate structures were also visible, but somewhat more diffused in yeast replicating the peroxisome-localized TBSV (Fig. 3A). The reduced localization of sterols in the plasma membrane was also noticeable in yeast replicating these tombusviruses.

To test if the redistributed sterols ended up in the membranes supporting tombusvirus replication, we performed co-localization experiments with RFP-tagged replication proteins and filipin dye to monitor sterol distribution. These experiments showed the enrichment of sterols in the subcompartment containing either CIRV p36 or the peroxisomal p33 replication proteins (Fig. 3B). We also performed experiments with fluorescently-labeled sterols (BODIPY-cholesterol) added to the growth media of yeast lacking the capacity to synthesize sterols (due to the deletion of the ergosterol biosynthesis *ERG9* gene) [65]. Interestingly, the fluorescently-labeled sterols were enriched in the same compartment as the tombusvirus p33 protein, or the CIRV p36, forming the characteristic punctate-like structures (Fig. 3C). Based on these data, we suggest that sterols (either synthesized internally in yeast or derived via intake from the culture media) are re-distributed and highly enriched at the sites of tombusvirus replication protein accumulation, which represent the sites of tombusvirus replication in yeast [12], [41].

### Sterols stimulate tombusvirus replication in artificial vesicles

Artificial vesicles (liposomes) formed from phosphatidylethanolamine (PE) can support *in vitro* replication of TBSV repRNA in the presence of viral replication proteins and the soluble fraction of CFE (Xu and Nagy, unpublished). We have tested if presence of sterols in the artificial PE vesicles could promote TBSV replication. The *in vitro* replication assay revealed that TBSV replication was increased to the highest extent (by up to ∼3-fold) when either ∼30% cholesterol or 10% ergosterol was present in the PE vesicles (Fig. 3D). Thus, the artificial vesicles-based assay supports the stimulatory role of sterols in tombusvirus replication.

### Tombusvirus p33 replication protein interacts with Scs2p VAP protein

The cellular ORPs are involved in non-vesicular lipid transfer and they are known to function at membrane contact sites (which are temporally formed areas where two membranes come to close vicinity) by facilitating the transfer of sterols and sterol derivatives from one membrane (the ER is usually the donor membrane) to the other membrane, serving as the acceptor membrane [54], [57], [58]. By hijacking the cellular ORPs, tombusviruses might be able to induce and/or stabilize the formation of MCSs to accelerate sterol transfer from the ER to peroxisomal membranes (or mitochondrial in case of CIRV). The MCS involving the ER membrane usually contains the ER-resident VAP proteins that facilitate the formation of MCSs [54], [57]. Indeed, we have identified the yeast Scs2p VAP protein in our previous genome-wide screens with tombusviruses [32], [35].

**Figure 4:**
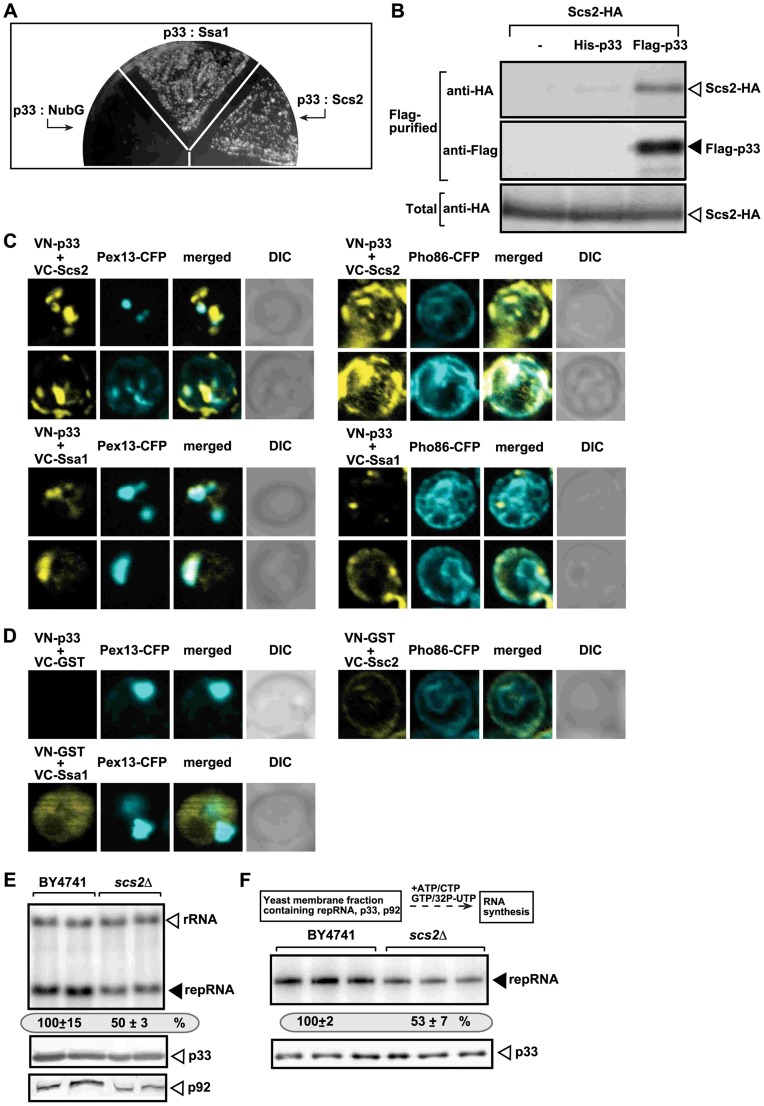
The tombusvirus p33 replication protein binds to the yeast Scs2p VAP protein in the ER. (A) The split ubiquitin assay was used to test binding between p33 and Scs2p in wt (NMY51) yeast. The bait p33 was co-expressed with the shown prey proteins. *SSA1* (HSP70 chaperone), and the empty prey vector (NubG) were used as positive and negative controls, respectively. (B) Co-purification of the Scs2p protein with the tombusvirus p33 replication protein. The FLAG-tagged p33 was purified from the membrane fractions of yeast extracts using a FLAG-affinity column. Top panel: Western blot analysis of co-purified 6xHA-tagged Scs2p using anti-HA antibody. Middle panel: Western blot of purified p33 (either His_6_- or Flag-tagged, as shown) detected with anti-FLAG antibody. Bottom panel: Western blot of 6xHA-tagged Scs2p in the total yeast extract using anti-HA antibody. (C) BiFC analysis of interactions between Scs2p and p33 and between Hsp70 (Ssa1p) and p33. Confocal laser microscopy images also show the peroxisomal localization of Pex13p marker protein (left panels) or Pho86-CFP ER-marker protein (right panels). The merged images at the top show the interaction between p33 and Scs2p and their partial co-localization with Pex13p-CFP or Pho86-CFP ER-marker, while the merged images at the bottom demonstrate the interaction between p33 and Ssa1 and their co-localization with Pex13p. DIC (differential interference contrast) images are shown on the right. Each row represents a separate yeast cell. Note that the Venus-(N-terminal portion) tag was fused to the N-terminus of p33 and the Venus-C tag was fused to host proteins, which are all N-terminal tags. Both p33 and Scs2p have cytosolic N-terminal regions. (D) Control BiFC experiments. Yeast was grown under similar conditions and images were taken as in panel C. (E) Decreased TBSV repRNA accumulation in *scs2Δ* yeast. To launch TBSV repRNA replication, we expressed His_6_-p33 and FLAG-tagged p92 from the copper-inducible *CUP1* promoter and DI-72(+) repRNA from the galactose-inducible *GAL1* promoter in the parental (BY4741) and in *scs2Δ* yeast strains. The yeast cells were cultured for 16 hours at 23°C on 2% galactose SC minimal media, and then for 24 h at 23°C on 2% galactose SC minimal media supplemented with 50 µM CuSO_4_. Northern blot analysis was used to detect DI-72(+) repRNA accumulation. The accumulation level of DI-72(+) repRNA was normalized based on 18S rRNA levels. Bottom panels: Western blot analysis of the accumulation level of His_6_-tagged p33 and FLAG-tagged p92 proteins using anti-His or anti-FLAG antibodies. Each experiment was performed three times. (F) Reduced activity of the tombusvirus replicase assembled in *scs2Δ* yeast. Top: Scheme of the experimental design. Denaturing PAGE analysis of *in vitro* replicase activity in the membrane-enriched fraction from wt and *scs2Δ* yeasts using the co-purified repRNA. The yeast cells were harvested for analysis at 24 h time point after launching TBSV replication. Note that this image shows the repRNAs made by the replicase *in vitro*. Each experiment was performed three times.

Therefore, to obtain evidence on the putative role of MCSs in tombusvirus replication, we studied the interaction between p33 replication protein and the yeast Scs2p VAP protein that is implicated in the regulation of lipid metabolism and transport. Scs2p is ER localized and binds to ORPs [53], [63]. Importantly, Scs2p is present at MCSs involving the ER [58]. We found that p33 replication protein strongly interacts with the yeast Scs2p based on membrane yeast two-hybrid assay (Fig. 4A) [33]. The interaction between the HA-tagged Scs2p and the FLAG-tagged p33 was confirmed using a co-purification assay based on FLAG-affinity purification of p33 replication protein from membrane fraction (Fig. 4B).

Using BiFC, we also show that the Scs2p: p33 interaction mostly takes place in the ER (Fig. 4C, top right panel), not in the peroxisome membrane (Fig. 4C, top left panel), where TBSV replication occurs, especially at the early stage of the replication process [41]. This is in contrast with the co-localization of Hsp70 (Ssa1p in yeast) and p33, which is mostly observed in the peroxisomal membrane (Fig. 4C, bottom panels), as demonstrated earlier [39]. The co-localization and interaction of p33 and Scs2p in the ER membrane are in agreement with the model that TBSV could interact with Scs2p and the Osh proteins to form/stabilize MCSs.

To study the relevance of p33: Scs2p interaction, we launched TBSV replication in *scs2Δ* yeast [59]. Interestingly, we found ∼50% reduction of TBSV replicon (rep)RNA level in *scs2Δ* yeast (Fig. 4E, top panel), suggesting that Scs2p is beneficial for TBSV replication. The p33 and p92 levels decreased in *scs2Δ* yeast (Fig. 4E, bottom panels), suggesting that Scs2p affects the stability of the tombusvirus replication proteins [46]. When we isolated the membrane-bound tombusvirus replicase from wt and *scs2Δ* yeast, we found ∼2-fold reduction in replicase activity *in vitro* after adjustment for comparable level of p33 replication protein in the replicase preparations (Fig. 4F). Overall, the inhibitory effect of *SCS2* deletion on TBSV replication, replicase activity and replication protein levels in yeast are similar to the effects caused by deletion of Osh3/5/6/7 in yeast (see above).

Expression of Scs2p from the strong *GAL1* promoter resulted in up to ∼8-fold increase in TBSV repRNA accumulation in *scs2Δ* yeast (Fig. S3), demonstrating that Scs2p is a positive host factor for TBSV. Moreover, complementation with the conserved VAP-motif containing MSP domain of Scs2p, which interacts with p33, also led to ∼5-fold stimulation of TBSV repRNA accumulation in *scs2Δ* yeast (Fig. S3), suggesting that the VAP region is critical to promote TBSV. These experiments established that two critical groups of cellular proteins, namely the ORP and VAP proteins, which are functional at MCSs, interact with the tombusvirus replication protein and stimulate tombusvirus replication.

### Over-expression of plant VAP proteins increases tombusvirus replication in yeast and plants

**Figure 5:**
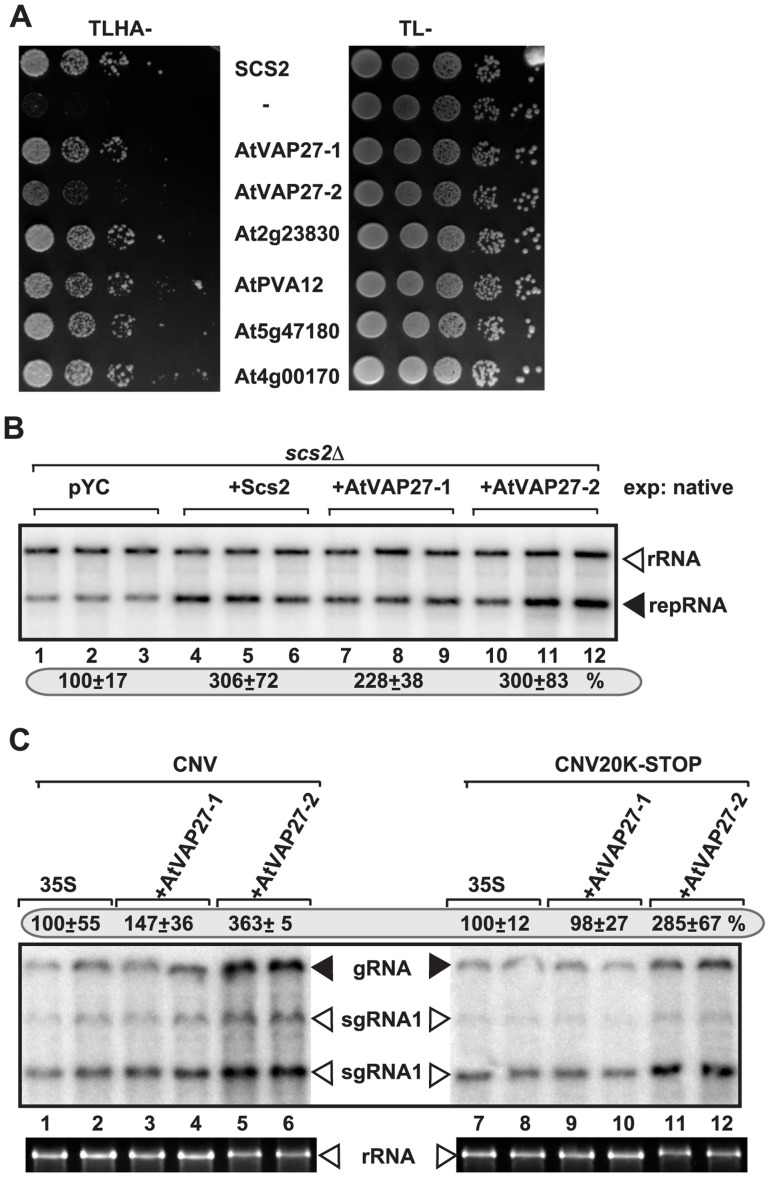
Six *Arabidopsis* VAP proteins interact with the TBSV p33 replication protein in yeast. (A) The split ubiquitin assay was used to test binding between p33 and six *Arabidopsis* VAP proteins in *scs2Δ* yeast. The bait p33 was co-expressed with the shown prey proteins. *SCS2* and the empty prey vector (NubG) were used as positive and negative controls, respectively. The left panel shows p33: VAP interactions, the right panel demonstrates that comparable amounts of yeasts were used for these experiments. (B) Expression of *Arabidopsis* VAP proteins can complement the defect in TBSV repRNA accumulation in *scs2Δ* yeast. Northern blot analysis of DI-72(+) repRNA accumulation in *scs2Δ* yeast expressing *Arabidopsis* VAP proteins from the native *SCS2* promoter. Note that pYC expresses a short peptide and used as a negative control. (C) Stimulation of tombusvirus RNA accumulation in plants by expression of two *Arabidopsis* VAP proteins. Expression of the *Arabidopsis* VAP proteins was done in *N. benthamiana* leaves, which were co-infiltrated with *Agrobacterium* carrying a plasmid to launch CNV replication from the 35S promoter. The control samples were obtained from leaves expressing no VAP proteins (35S, lanes 1–2 and 7–8). Total RNA was extracted from leaves 3 days after agroinfiltration. The accumulation of CNV RNAs in *N. benthamiana* leaves was analyzed by Northern blot. The ribosomal RNA (rRNA), visualized by ethidium-bromide staining, was used as a loading control.

Although over-expression of ORPs in yeast or plants had limited effect on tombusvirus replication (likely due to their abundance and/or redundant nature) (not shown), we attempted over-expression strategy with plant VAP proteins, which are conserved among eukaryotes [59]. First, we showed that six different *Arabidopsis* VAP proteins could interact with TBSV p33 protein in the yeast split-ubiquitin assay (Fig. 5A), similar to the interaction between p33 and the yeast Scs2p (Fig. 5A). Then, we demonstrated that expression of AtVAP27-1 and AtVAP27-2 proteins [66], [67] increased TBSV repRNA accumulation in *scs2Δ* yeast by up to ∼3-fold (Fig. 5B, lanes 7–12), suggesting that these plant VAP proteins can stimulate TBSV replication.

Over-expression of AtVAP27-2, and to a lesser extent AtVAP27-1 proteins in *Nicotiana benthamiana* leaves also increased the accumulation of *Cucumber necrosis virus* (CNV) genomic RNA, which is very closely related to TBSV, by up to ∼3.5-fold (Fig. 5C, lanes 7–12). Based on these experiments, we suggest that the plant VAP proteins likely play similar roles in tombusvirus RNA replication to the yeast Scs2p VAP protein. The plant VAP proteins are important for the recruitment of plant ORP proteins to the ER membrane, suggesting similar functions for these cellular proteins to their counterparts in yeast and animals [56].

**Figure 6:**
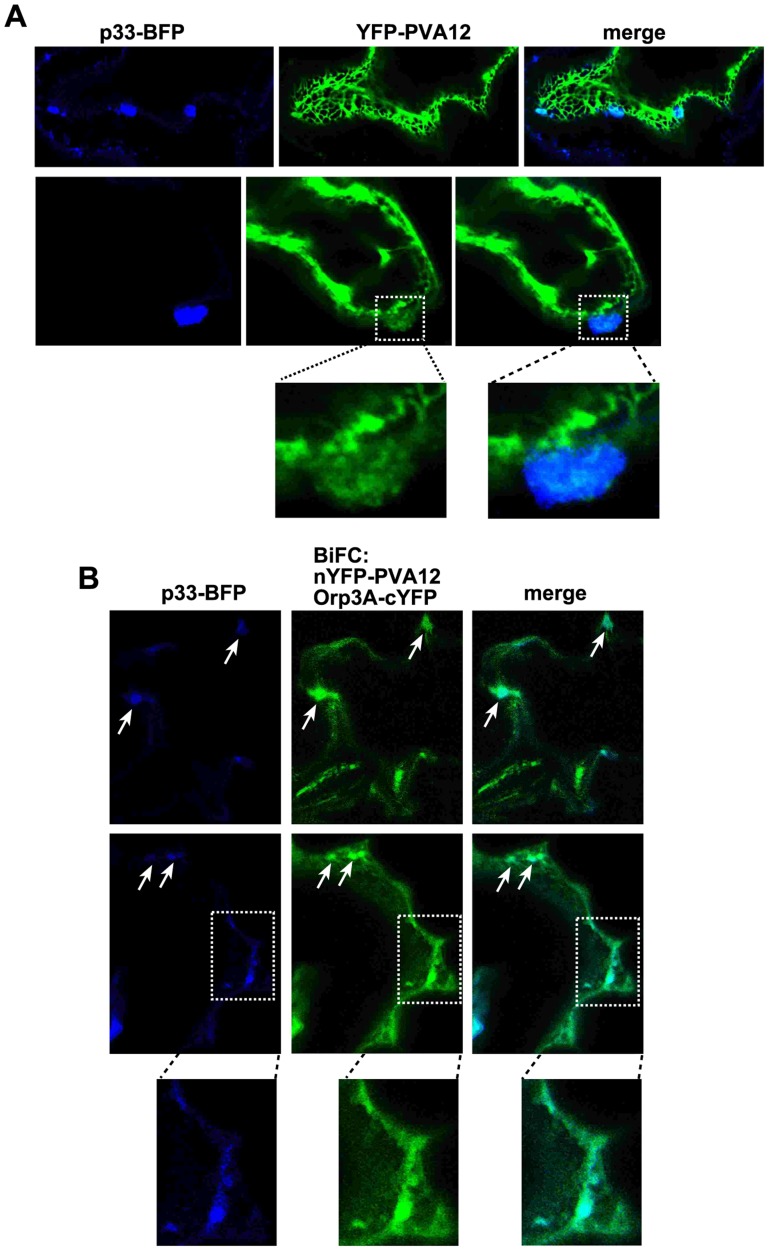
The tombusvirus p33 replication protein co-localizes with AtOrp3A and AtPVA12 proteins in *Nicotiana benthamiana*. (A) Partial co-localization of TBSV BFP-tagged p33 replication protein with the YFP-AtPVA12 VAP protein in *N. benthamiana* cells. Expression of the above proteins from the 35S promoter was done after agro-infiltration into *N. benthamiana* leaves. Note the altered membrane structure in the subcellular area showing co-localization of BFP-p33 and YFP-AtPVA12 (portion of the image was enlarged at the bottom panel), which might be due to local membrane proliferation. (B) Co-localization of TBSV BFP-tagged p33 replication protein with the nYFP-AtPVA12 VAP and AtOrp3A-cYFP protein complexes in *N. benthamiana* cells. Expression of the above proteins from the 35S promoter was done after agro-infiltration into *N. benthamiana* leaves. Note that nYFP-AtPVA12 VAP and AtOrp3A-cYFP proteins were detected by BiFC. The subcellular areas (likely representing the ER membranes) where one viral and two cellular proteins are co-localized are marked with arrows. Control BiFC experiments were as in a previous paper (not shown) [56].

To further demonstrate the similarity between yeast and plant regarding the roles of ORPs and VAPs in TBSV replication, we used confocal laser microscopy to analyze the subcellular localization of BFP-tagged p33 and YFP-AtPVA12 in *N. benthamiana* leaves. Interestingly, a portion of BFP-p33 molecules is co-localized with YFP-AtPVA12 (Fig. 6A). In addition, BFP-p33 is also partially co-localized with AtOrp3A and AtPVA12 complexes (detected by BiFC, Fig. 6B). Altogether, the *in planta* data suggest comparable distribution and co-localization of viral p33 replication protein in relation with plant ORP and VAP proteins as observed in yeast.

### Formation of tombusvirus-induced spherules close to membrane contact sites in plants and yeast

**Figure 7:**
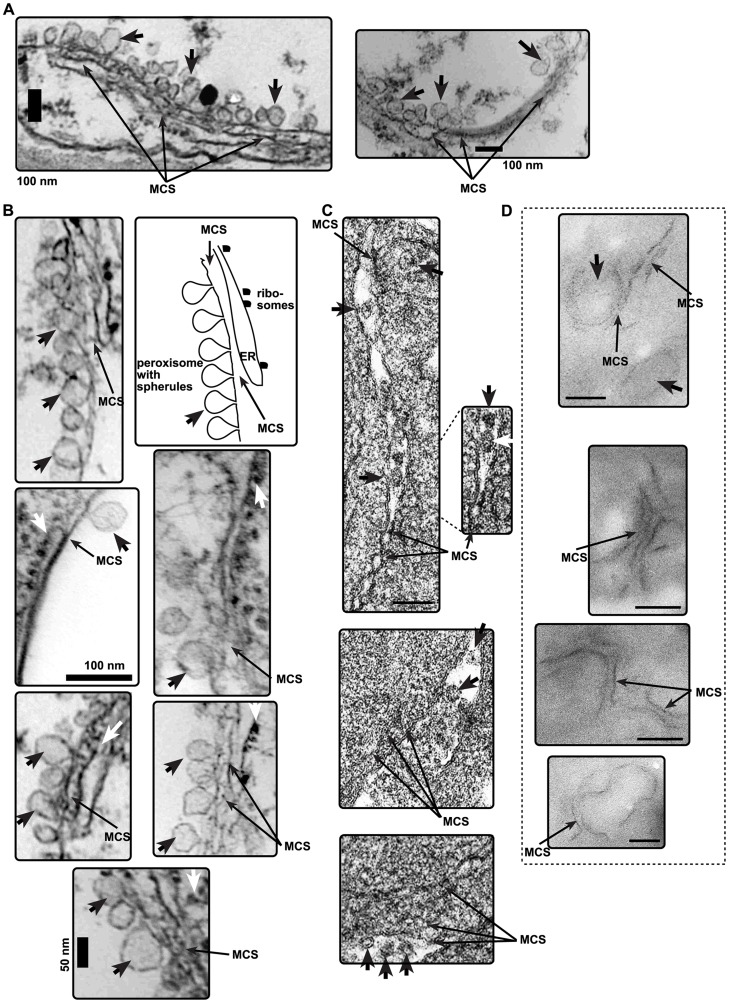
The presence of MCS-like structures in the vicinity of tombusvirus-induced spherules in plant cells infected with CNV or in yeast cells replicating TBSV repRNA. (A) Representative electron microscopic images of portions of *N. benthamiana* cells. Several characteristic virus-induced spherules are marked with arrowheads and the MCS-like structures are indicated by arrows. These spherules are formed via membrane invagination into peroxisome-derived membranes. (B) Close up view of spherules and MCS-like structures in plant cells infected with CNV. The ER and the associated ribosomes are indicated with white arrowheads. See further details in panel A. (C) TEM of stained ultra-thin sections in yeast cells replicating TBSV repRNA. Characteristic spherule-like vesicles are assembled in a peripheral membranous compartment (arrows) in the vicinity of MCS-like structures. (D) METTEM of ultra-thin sections of yeasts replicating TBSV repRNA. The MT-tagged p33 was visualized by ∼1 nm gold nano-clusters associated to MT. Black arrowhead points at a spherule-like structure, while black arrows show possible MCS with p33 present in opposing membranes. Bars in panels C-D represent 100 nm.

By co-opting the cellular ORP and VAP proteins via interaction with the viral replication protein, tombusviruses likely induce and/or stabilize the formation of MCSs to accelerate sterol transfer from the ER to the sites of replication. To study the predicted formation of MCSs during viral replication, we used EM to visualize the tombusvirus-induced spherules (vesicle-like structures with narrow openings toward the cytosol), which represent the sites of viral RNA replication in tombusvirus-infected plants. Indeed, we frequently observed additional membranes in the close vicinity (separated by 10–30 nm distance) of the peroxisomal membranes containing the characteristic ∼50–70 nm tombusvirus-induced spherules (Fig. 7A). Higher magnification of these membranes indicates that ER sacks (with some ribosomes) are juxtaposed to the peroxisomal membrane (Fig. 7B). Additional EM images with either the genomic RNA or the DI-72 replicon RNA further supported the frequent occurrence of juxtaposed membranes, thus the formation of MCS-like structures, close to viral spherules in plants (Fig. S4). We also observed MCS-like structures in the vicinity of viral spherules in EM images of yeast cells expressing the viral replication proteins and the TBSV repRNA (Fig. 7C). Thus, these EM structures support the model that MCSs are formed between subcellular membranes in the vicinity of the tombusvirus-induced spherules in plant cells.

To detect if p33 replication proteins are present in structures similar to MCSs, we used Metal-Tagging Transmission Electron Microscopy (METTEM) [68], [69]. The METTEM-based imaging of yeast cells expressing MT-tagged p33 revealed the presence of juxtaposed elongated p33 clusters that resembled MCSs (Fig. 7D and S5B). Distribution of MT-p33 in these METTEM images indicates that p33 is likely present both in the donor and acceptor membranes at MCSs (Fig. 7D). In several images, these MCS-like structures were in the vicinity of spherules (visible as more round-shaped structures). Gold-labeled antibody-based detection of dsRNA, which is present in the tombusvirus VRCs, revealed that the viral dsRNA is unlikely being made at the MCSs, but instead, in the VRCs that are detected as more globular structures by METTEM (Fig. S5A and C). Altogether, the obtained data with METTEM suggest that p33 replication protein could be present in both membranes (donor and acceptor) in the putative MCSs.

## Discussion

Lipids and subcellular membranes are critical for (+)RNA viruses in order to assemble membrane-bound VRCs or form viral replication organelles during their replication in infected cells [5], [18], [70]. The VRC assembly depends on reshaping/deforming membranes to generate membranous structures, such as spherules and vesicles. In addition, many (+)RNA viruses also induce membrane proliferation by enhancing the synthesis of new lipids, or by redirecting lipids to the sites of viral replication [71]–[74]. Among the targeted lipids are phospholipids and sterols, which are major components of cellular membranes, affecting the size, shape, curvature and rigidity of membranes and intracellular organelles [75]. Therefore, not surprisingly, many genome-wide screens performed with (+)RNA viruses have led to the identification of a number of host genes affecting lipid biosynthesis or metabolism [8], [10], [76], [77].

**Figure 8:**
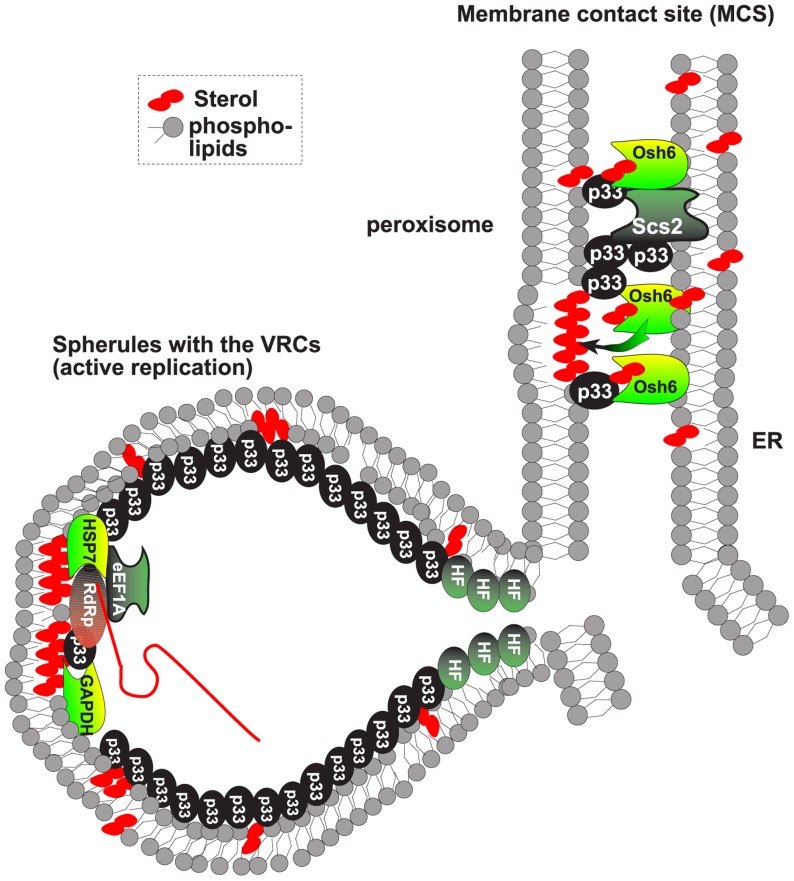
Model on the role of ORP and VAP proteins and MCSs in tombusvirus replicase assembly. The ORP proteins, such as the yeast Osh6p, are recruited via binding to the tombusviral p33 replication protein and the ER-bound Scs2p VAP to MCS, formed between the ER and peroxisomal membranes. The ORPs then facilitate the enrichment of sterols in the peroxisomal membrane (or mitochondrial membrane in case of CIRV), forming sterol-rich microdomains needed for the formation of virus-induced spherules. These spherules contain the VRCs performing viral RNA synthesis. Note that the MCSs are not part of the VRCs based on the difference in BiFC localization of p33:Ssa1p Hsp70 complex and p33:Scs2p, which suggests that different subpopulations of p33 are involved in each interaction. We have previously shown that the Ssa1p is present in the active VRCs.

In this paper, we show that members of lipid transfer proteins, namely the oxysterol-binding ORP proteins are recruited by tombusviruses to the sites of viral replication. The direct binding of Osh3/5/6/7 to the tombusvirus p33 replication protein leads to the enrichment of these ORPs in peroxisomal (or in mitochondrial in case of CIRV) and ER subcompartments. This also leads to enrichment of sterols at the site of viral replication. We propose that the subverted ORP proteins likely facilitate the re-distribution of sterols to viral sites (Fig. 8) at the expense of their natural plasma membrane localization. The enrichment of sterols in the membranes around the replicase complex likely promotes viral RNA replication as we observed more efficient TBSV repRNA replication in artificial PE vesicles containing cholesterol or ergosterol.

The obtained data support the functional role of the cellular ORP proteins as vesicle-independent sterol transporters in tombusvirus replication. This is because combined deletion of *OSH3*, 5, 6 and 7 resulted in similar debilitating changes in viral replication to that observed by perturbing sterol metabolism via down regulation of *ERG25* or deletion of *ERG4* sterol biosynthesis genes in yeast model host [7], [47]. These changes included (i) major reduction in tombusvirus replication; (ii) instability of viral replication proteins; and (iii) the low efficiency of tombusvirus replicase assembly/activity. It is plausible that the enrichment of sterols around the viral replication proteins via recruitment of ORP proteins facilitates virus-induced membrane deformation and the generation of viral spherules that represent the sites of tombusvirus replication (Fig. 8) [12]. In the absence of ORP proteins, low concentration of sterols around the membrane-bound replication proteins could also hinder protein-protein interactions and lead to degradation of viral replication proteins not protected by the spherule-like structures.

Tombusviruses likely exploit ORP proteins at MCSs to enrich the sterols in subdomains of either peroxisomes (in case of TBSV) or mitochondria (CIRV). Indeed, the yeast Osh proteins and other eukaryotic ORPs are active in directional sterol transport when membranes of various organelles form temporal MCSs [54], [57], [58]. This model is supported by the observation that Osh6p : p33 replication protein complexes were co-localized to both peroxisomal and ER membranes, while the CIRV p36 : Osh6p complexes were localized to both mitochondrial and ER membranes (Fig. 2). Moreover, the ER-localized p33 : Scs2p complexes were observed in close vicinity of peroxisomal membranes (Fig. 4). Further evidence on the proposed role of MCSs in VRC/spherule formation during tombusvirus replication was obtained by EM images of tombusvirus-infected plant cells or yeast cells supporting TBSV replication (Fig. 7). The TBSV-induced spherules were frequently located in the vicinity of ER-like membrane sacks, suggesting the presence of MCSs at or in the vicinity of the site of tombusvirus replication.

Another supporting evidence for the role of MCSs is the interaction between the tombusvirus p33 replication protein and the yeast Scs2p VAP protein in the ER membrane. The interactions between Osh proteins and Scs2p are known to promote the formation of MCSs involving the ER membrane [54], [57], [58]. We found that the p33 replication protein interacts with both Scs2p VAP protein and ORPs (i.e., Osh3/5/6/7), indicating that tombusviruses might enhance sterol transfer to the site of replication via stabilizing MCSs to aid the formation of tombusvirus-induced spherules and VRCs (Fig. 8). Based on the observed interactions and intracellular localization data, it is likely that both peroxisome (in case of TBSV and CNV) and mitochondria-based (for CIRV) replication depends on MCSs in host cells.

ORPs and VAP sequence containing proteins similar to the yeast Scs2p also exist in plants, suggesting that comparable strategy involving cellular VAP proteins and ORPs could be used by tombusviruses in their native hosts. Accordingly, we show that the TBSV p33 replication protein interacts with six *Arabidopsis* ORPs and five different VAP proteins and expression of two of these VAP proteins in *scs2Δ* yeast increased TBSV repRNA accumulation. Moreover, over-expression of one of these *Arabidopsis* VAP proteins also increased tombusvirus genomic RNA accumulation in *N. benthamiana* leaves (Fig. 5C), indicating that the host VAP proteins are also beneficial to tombusvirus replication in plants. Moreover, partial co-localization of BFP-p33 molecules with YFP-AtPVA12 and p33 or peroxisomes (only in TBSV-infected cells) with AtOrp3A and AtPVA12 complexes (Fig. 6) suggests comparable roles of co-opted ORPs and VAPs in TBSV replication in yeast and plants.

Sterols not only affect tombusviruses, but replication of other RNA viruses, such as Dengue virus, Norwalk virus, hepatitis C virus (HCV) and picornaviruses [74], [78]–[81]. Interestingly, the HCV replicase complex is associated with cholesterol-rich lipid rafts that could promote high local concentration of viral proteins [82]. West Nile virus has been shown to induce the redistribution of cholesterol to the sites of virus replication, possibly from the plasma membrane [83]. The reduced sterol level in the plasma membrane likely inhibits antiviral responses, the entry process of animal viruses to cells, and the exit of virus particles from cells [84]–[87]. It will be interesting to see if lipid-transfer ORP proteins, VAP proteins and MCSs are involved in the infection process of other viruses and pathogens.

### Summary

In this paper, we document that selected ORPs and VAP proteins are co-opted by tombusviruses. We propose that viral subversion of these host proteins leads to enrichment of sterols at the sites of tombusvirus replication likely via MCS formation. The increased amount of sterols in viral protein-containing microdomains is proposed to facilitate the formation of spherules and vesicle-like structures that are needed for tombusvirus replication.

## Materials and Methods

### Analysis of tombusvirus replication in yeast

Yeast strains SEY6210 and JRY6266 *his3*Δ (see plasmids used in Materials and Methods S1) were transformed with plasmids pESC/DI72/His33 (or pESC/DI72/His36), pGAD(Trp1)-His92-CUP1 (or pESC(Trp1)-Flag95-CUP1) and pYC2/NT-C plasmids expressing one of the His_6_-tagged Osh proteins. Yeast strains SEY6210, JRY6266, JRY62323 and JRY6207 were transformed with plasmids pESC(Ura3)/DI72/His33 and pGAD(Trp1)-His92-CUP1. SEY6210 and JRY6259 were transformed with pESC(Trp1)/DI72/His33 and pGAD-His92-CUP1. Transformed yeasts were pre-grown in SD minimal media supplemented with 2% glucose for 24 hours at 29°C [88]. Then, the yeast cultures were centrifuged, washed in 2% galactose minimal media and used to start cultures in 2% galactose minimal media plus 50 µM CuSO_4_ (starting with 0.25 OD_600_). Cultures were grown for 48 hours at 29°C.

Yeast strains BY4741 and *scs2Δ* were transformed with pGBK-His33-CUP1/DI72-GAL1, pGAD-flag92-CUP1 and pYC2/NT-C plasmids expressing one of the Scs2/VAP proteins. Transformed yeasts were grown in SD minimal media supplemented with 2% galactose for 20 hours at 29°C, then supplemented with 50 µM CuSO_4_ and grown for an additional 24 hours. Total RNA extractions and Northern blot analyses were performed as described previously [21], [88]. DI-72 (+)repRNA was detected with a ^32^p-labelled RNA probe and the relative accumulation determined using the 18s rRNA for normalization [89].

Accumulation of the viral replication proteins was determined by Western blot using anti-His or anti-Flag antibodies followed by anti-mouse conjugated to alkaline phosphatase and NBT/BCIP for detection [89].

### Analysis of TBSV replication in cell-free extracts

Cell-free extracts (CFE) from yeast strains SEY6210, JRY6266 and JRY6259 were prepared as described previously [26]. MBP-tagged p33 and p92 proteins were purified from *E. coli* as described [90]. CFE-based reaction mixtures containing 0.1 µg of each purified protein, 0.5 µg *in vitro*-transcribed repRNA and CFE in a 20 µl final volume were incubated at 25°C for 3 hours. The amount of newly synthesized repRNA was analyzed in polyacrylamide/Urea gels as described [62].

### Analysis of tombusvirus replication in plants

*Nicotiana benthamiana* leaves were co-infiltrated with *Agrobacterium tumefaciens* carrying the plasmid pGD-CNV or pGD-CNV20k-stop [12] and *A. tumefaciens* carrying pGD-L plasmids expressing *A. thaliana* VAP27-1 or VAP27-2 proteins. Total RNA was extracted from the infiltrated leaves 3 days after agro-infiltration and the accumulation of CNV genomic RNA was analyzed by agarose electrophoresis as described [12].

### Analysis of protein interactions in vivo using yeast membrane two-hybrid assay

The yeast membrane two-hybrid assay, based on the split-ubiquitin system has been described before [35]. The plasmid pGAD-BT2-N-His33 was co-transformed with pPR-N-RE derived constructs into yeast strain NMY51 (Dualsystems). Transformed yeasts colonies were suspended in 100 µl of water and 5 µl of the suspension spread onto TL^−^ plates, as loading control, or onto TLHA^−^ plates to score protein interactions [35] and incubated at 30°C. Alternatively, colonies were suspended in 100 µl of water and serially diluted (by 10-fold) in water. 6 µl of each dilution were spotted onto TL^−^ or TLHA^−^ plates.

### Co-purification of yeast Osh proteins with Flag-p33 from cellular membranes

For co-purification of chromosomally expressed 6xHA-tagged proteins with tombusvirus FLAG-p33 replication protein, yeast strains OSH6-6xHA, OSH7-6xHA and SCS2-6xHA were transformed with plasmids pGBK-His33-CUP1/DI72-GAL1 or pGBK-Flag33-CUP1/DI72-GAL1 and pGAD-His92-CUP1. For co-purification of His_6_-tagged proteins with CNV p33, yeast strain BY4741 was co-transformed with plasmids pESC/DI72/His33 or pESC/DI72/Flag33, pGAD-His92 and pYC2/NT-C plasmids expressing one of the His_6_-tagged Osh proteins. For co-purification of His_6_-tagged proteins with CIRV p36 replication protein, yeast BY4741 was co-transformed with plasmids pESC/DI72/His36 or pESC/DI72/Flag36, pESC(Leu2)-Flag95-CUP1 and pYC2/NT-C plasmids.

Transformed yeasts were pre-grown for 24 hours at 29°C in SD media containing 2% glucose. After centrifugation, the pre-grown yeast was used to inoculate 50 ml SD media supplemented with 2% galactose plus 50 µM CuSO_4_ and grown for another 24 hours at 29°C. The cultures were centrifuged, washed with phosphate buffer saline (PBS) and incubated in PBS plus 1% formaldehyde for 1 hour on ice to crosslink proteins [12]. Formaldehyde was quenched by addition of glycine (to 0.1 M) and the yeast was recovered by centrifugation. FLAG-p33 was purified from cellular membranes using anti-FLAG M2 agarose as described previously [35]. Purified FLAG-p33 was analyzed by Western blot using anti-FLAG antibody followed by anti-mouse antibody conjugated to alkaline phosphatase [35]. Co-purified His_6_-tagged Osh proteins were analyzed with anti-His antibody followed by anti-mouse antibody conjugated to alkaline phosphatase. Chromosomally expressed Osh6-6xHA, Osh7-6xHA, and Scs2-6xHA were analyzed with anti-HA antibody followed by anti-rabbit antibody conjugated to alkaline phosphatase [12]. Detection with NBT-BCIP was done as described [12], .

### *In vitro* protein interaction assays

MBP-tagged TBSV p33 and its truncated derivatives were expressed in *E. coli* and purified in amylose columns as described [90]. Lysates of *E. coli* expressing recombinant GST-His_6_-Osh proteins were then passed through the amylose columns containing the captured MBP-p33 protein derivatives. After washing, MBP-p33 proteins were eluted with maltose. The amount of bound GST-His_6_-Osh proteins was analyzed by SDS-PAGE and Western blot using anti-His antibody as described [92].

### Bimolecular fluorescence complementation (BiFC) assay in yeast

The ORF for the N-terminal portion of Venus fluorescent protein comprising amino acids 1 to 155 was PCR-amplified with primers #3907/#3762, digested with *Nco*I and *Bam*HI and ligated into *Nco*I/*Bam*HI-digested pESC/DI72/His33 [93]. The resulting plasmid, pESC-VenN-p33-DI72, expresses the N-terminal portion of Venus (aa 1–155) fused to the p33 protein. The *Nco*I/*Bam*HI-digested Venus-N product was also ligated into *Nco*I/*Bam*HI-digested pESC/DI72/His36 to generate pESC-VenN-p36-DI72. The sequence for the C-terminal portion of Venus (aa 155–236) was PCR-amplified with primers #3763/#3764, digested with *Hind*III and *Bam*HI and ligated into *Hind*III*/Bam*HI-digested pYC2/NT-C. *OSH6, SCS2 and SSA1* sequences were PCR-amplified with primers #5569/#5132, #2990/#2991 and #2653/#2812, respectively, digested with *Bam*HI and *Xho*I and ligated into *Bam*HI/*Xho*I-digested pYC-VenC. pESC-VenN-p33-DI72 or pESC-VenN-p36-DI72 in combination with pYC-VenC-OSH6 and pGAD-pho86-CFP, pGAD-pex13-CFP or pGAD-ilv6-CFP [40] were co-transformed into Sc1 yeast strain. Transformed yeast colonies were grown in liquid SD media containing 2% galactose for 24 hours at 29°C and confocal laser microscopy was performed as previously described [40].

Co-localization and Bimolecular Fluorescence Complementation Assay *in planta*. pGD-35S-p33-BFP plasmid was created by PCR amplification of CNV p33 ORF with primers #424F and #3570R. Fluorescent protein TagBFP was amplified with primers #5877F and #5878R using TagBFP-AS-N plasmid (Evrogen) as a template. The PCR products were digested with *Nhe*I and *Xba*I respectively, ligated and a second PCR was performed with primers #424F and #5878R. The PCR product was digested with *BamH*I and *Sal*I and then ligated into pGD-35S (the original was provided by M. Goodin) vector digested with the same enzymes. pGD-35S-BFP_SKL_ plasmid was constructed by ligating the PCR product amplified with primers #5877F and #5879R into pGD-35S vector via *BamH*I and *Sal*I restriction sites. The plasmids were transformed into *Agrobacterium* strain C58C1Rif. Plasmids, harboring YFP-PVA12, nYFP-PVA12 and ORP3a-cYFP were used as described earlier [56]. Four weeks old *N. benthamiana* leaves were agro-infiltrated for the transient expression of the tagged proteins. Transformed leaves were analyzed 48 h after agro-infiltration and confocal laser imaging was performed using an Olympus FV1000 microscope as described previously [94].

### Filipin staining of sterols and confocal microscopy

Sc1 yeast strain was co-transformed with plasmids pYC-RFP-p33, pGAD-His92 and pGBK-DI72 or pYC-RFP-p36, pESC(Leu2)-Flag95-CUP1 and pGBK-DI72. Transformed yeasts were grown in SD minimal media supplemented with 2% galactose and plus 50 µM CuSO_4_ for 24 hours at 29°C. Cells were fixed and stained with filipin as described [95]. Briefly, yeast cultures were treated with formaldehyde (3%) for 15 minutes to fix cells. Then, cells were centrifuged, washed with water 2 times and finally re-suspended in 1 ml of water. 20 µl of filipin solution (5 mg/ml in ethanol) was added, followed by incubation in the dark for 15 minutes. Then 3 µl of the cell suspensions were directly spotted onto poly-lysine microscope slides and examined in a confocal microscope (405 nm laser for filipin and 543 laser for RFP) or in a UV light microscope (Zeiss) using a DAPI filter set.

### Lipid vesicles preparation

1,2-dioleoyl-*sn*-glycero-3-phosphoethanolamine (PE, 18∶1), L-α-phosphatidylinositol-4-phosphate (PI4P, from brain), Cholesterol and Ergosterol were purchased from Avanti Polar lipids, Inc., dissolved and stored in chloroform. In each preparation, 269 nmol PE plus additional PE, PI4P or sterols were added into glass vial, mixed, and subsequently dried by a gentle stream of nitrogen for 1 hour, and further dried in speed-vacuum for 2–3 hours. 400 µl HEPES buffer (30 mM HEPES-KOH, pH 7.4) was added to each vial, and the preparations were subjected to sonication in a bath sonicator (Avanti Polar lipids, Inc.) filled with icy water for about 20 minutes, until the mixture become visually homogeneous. Lipid vesicles were used on the day of preparation for *in vitro* replication assay.

### *In vitro* TBSV replication assay

The experimental procedure for *in vitro* replication assay using phospholipid vesicles was same as previously published procedure using purified yeast organelles [96], except that a 40,000 g supernatant (S40) from yeast cell-free extract was used, and lipid vesicles were used as membrane source instead of purified yeast organelles.

### Fluorescence cholesterol labeling of yeast cells

Sterol auxotroph *Saccharomyces cerevisiae erg9*Δ strain [65] (MATa *his3Δ, leu2Δ, met15Δ, ura3Δ, erg9Δ*::hphNT1 derivative, kindly provided by Dr. Joe Chappell, UKY, USA) was transformed with plasmids expressing mRFP tagged CNV or CIRV replication proteins p33 or p36 [96]. Transformed yeast cells were pre-cultured overnight in synthetic complete dropout (SC) media containing 20 mg/L cholesterol, and diluted into SC media with galactose supplemented with 10 mg/L BODIPY-cholesterol (TopFluor Cholesterol, Avanti polar lipids, Inc.) at 0.5 OD_600_. The yeasts were incubated at 23°C for 16 hours and then subjected to confocal laser microscope analysis [40].

### Electron microscopy

Methods have been previously described in details [12], [15]. Briefly, *N. benthamiana* leaves were agro-infiltrated with a construct expressing CNV 20 k stop or a combination of *A. tumefaciens* cultures expressing p33, p92, p19 proteins (viral suppressor of gene silencing), and DI-72 RNA. 2.5 days after agro-infiltration, leaf samples were incubated with a fixing buffer containing 0.1 M KPO4, pH 6.8, 3.5% glutaraldehyde and 1% paraformaldehyde. The leaves were injected with the fixing buffer using a syringe (without needle) and subsequently sectioned into 1×5 mm strips. The leaf sections were immersed in the fixing buffer and incubated overnight at 4°C. Leaf sections were washed three times for 10 minutes in 0.1 M KPO4 pH 6.8, plus 5% glucose, then treated with 1% OsO_4_ for 2 hours at room temperature (RT). Sections were washed in distilled water for 5 min and dehydrated sequentially in 50%, 70%, 80% and 90% ethanol for 10 min each at RT, followed by two incubations with 100% ethanol for 20 min and two with propylene oxide (PO) for 15 min. Samples were gradually infiltrated in 50/50 epon-araldite resin/PO overnight, 75/25 resin/PO for 4 hours and then 100% resin for 4 hours under vacuum. Samples were finally embedded in pure resin and incubated for 48 hours at 60°C for resin polymerization. After sectioning and mounting in copper grids, samples were stained with uranyl-acetate and lead-citrate and imaged in a Philips Biotwin12 transmision electron microscope. The images were cropped using Photoshop software.

For electron microscopy of yeast cells, the yeast BY4741 strain was pre-cultured from plated single colonies by inoculation in 2 ml of YPG (yeast extract peptone galactose). The culture was then incubated at 30°C and shaken overnight at 250 rpm. For inducing and maintaining viral replication, yeasts cells were grown in YPG at 23°C and shaken at 250 rpm for 24 h. When OD_600_ was around 2, cells were centrifuged for 5 min at 4000× g, re-suspended in TSD reduction buffer (Tris-sulfate DTT, pH 9.4) and treated for removing the cell wall to obtain spheroplasts. Compared to whole yeast cells, spheroplasts are well infiltrated with fixatives and resins allowing an optimal preservation and visualization of cell ultrastructure. For obtaining spheroplasts, yeast cells were maintained for 10 min at room temperature (RT) and then treated at 30°C with 0.1 µg/µl zymolyase 20T (AMS Biotechnology) in spheroplast medium A (1× yeast nitrogen base, 2% (w/v) glucose, 1× amino acids, 1 M sorbitol, 20 mM TrisCl, pH 7.5) for 5 or 15 min, depending on the yeast strain. After zymolyase treatment, cells were centrifuged for 5 min, at 1000 g and 23°C and washed once with spheroplast medium B (1× yeast nitrogen base, 2% (w/v) glucose, 1× amino acids, 1 M sorbitol) and twice with spheroplast medium A. Cells were chemically fixed in two steps: 1) 20 min in suspension at RT with 8% paraformaldehyde and 1% glutaraldehyde and 2) 1 h at RT with 4% paraformaldehyde and 0.5% glutaraldehyde in HEPES (pH 7.4); fixed cells were then processed by conventional embedding in the epoxy-resin EML-812 (Taab Laboratories). The embedding protocol, designed to provide an optimal preservation of cell membranes, was previously described in detail [97]–[99]. In brief, cells were post-fixed for 1 h at 4°C with 1% osmium tetroxide and 0.8% potassium ferricyanide in water, washed with HEPES, and incubated 40 min with 2% uranyl acetate at 4°C. Dehydration was done at 4°C with increasing concentrations of acetone (50, 70, 90, and twice in 100%, 10 min each). Samples were then incubated at room temperature with a mixture of acetone and resin (1∶1). Cells were infiltrated with 100% resin for 1 day and polymerized at 60°C for 3 days. Ultrathin (50–70 nm) sections were collected in formvar-coated 300 mesh cooper grids (G300-C3, Taab). Sections on grids were stained with saturated uranyl acetate and lead citrate following standard procedures and studied in a Jeol JEM 1011 transmission electron microscope operating at 100 kv.

For visualization of MT-tagged p33, we processed the yeast strain DB-614 (BY4741 *ADH1*p-His-p92::*kanMX4*, *GAL1*p-His-MT-p33/*GAL10*p-DI72::*hphNT1*) expressing His_6_-p92 under control of the *ADH1* promoter and *ADH1* terminator [100], and the metallothionein 1 (MT-1)-tagged p33 (His6-MT-p33) under control of *GAL1* promoter and *CYC1* terminator, and DI-72 under control of *GAL10* promoter and *ADH1* terminator [15]. For building gold nano-clusters in MT-p33 molecules, these yeast cells were incubated with gold salts and processed for embedding in the acrylic resin LRWhite [15]. The embedding protocol allowed an adequate preservation of protein epitopes and the optimal visualization of the small gold nano-clusters [68]. Spheroplasts were incubated for 75 min with 0.2 mM HAuCl_4_ (SIGMA-ALDRICH) in spheroplast medium A. This treatment builds gold nano-clusters in MT-tagged proteins allowing detection of protein molecules in cells with high sensitivity and at molecular scale resolution [68], [69]. Cells were washed with spheroplast medium A and fixed 1 h at RT with 4% paraformaldehyde and 0.2% glutaraldehyde in PHEM (20 mM PIPES, 50 mM HEPES, 20 mM EGTA and 4 mM MgCl_2_, pH 6.9). After short dehydration steps of 10 min each in increasing concentrations of ethanol (30, 50, 70, 90 and twice with100%) at 4°C, spheroplasts were incubated in mixtures of ethanol and LR White (2∶1, 2∶2, 1∶2) and in 100% resin for 24 h. For resin polymerization samples were maintained at 60°C for 48 h. Ultra-thin sections were collected in 300 mesh Quantifoil holey carbon grids (R 3.5/1 Cu/Rh, Quantifoil Micro Tools) and studied without staining. For immunogold labeling of dsRNA, sections were incubated for 6 min with 1% bovine serum albumin (BSA) in PBS, with an anti-dsRNA primary antibody diluted 1∶200 in 1% BSA and with secondary antibodies conjugated with 5 or 10 nm colloidal gold particles and diluted 1∶40 in 1% BSA. The mouse anti-dsRNA MAb J2 was from English & Scientific Consulting and the secondary antibodies conjugated with colloidal gold particles were from BB International.

## Supporting Information

Figure S1 **Identification of the yeast Osh proteins interacting with the tombusvirus p33 replication protein.** (A) Co-purification of the yeast Osh3p, Osh5p and Osh6 proteins with the CNV p33 replication protein. Top panel: Western blot analysis of co-purified His_6_-tagged cellular Osh proteins with Flag-affinity purified p33 from membrane fraction. The Osh proteins were detected with anti-His antibody. The negative control was His_6_-tagged p33 purified from yeast extracts using a FLAG-affinity column. Bottom panel: Western blot of purified Flag-p33 detected with anti-FLAG antibody. Western blot of His_6_-tagged Osh1-6 proteins in the total yeast extracts using anti-His antibody. (B) Affinity binding assay to detect interaction between His_6_-tagged Osh and the MBP-tagged TBSV p33 protein (the C-terminal portion). The MBP-tagged viral protein or MBP control produced in *E. coli* was immobilized on amylose-affinity columns. Then, His_6_-tagged Osh3, 5, 6, 7 proteins expressed in *E. coli* were passed through the amylose-affinity columns with immobilized MBP-tagged proteins. The affinity-bound proteins were eluted with maltose from the columns. The eluted proteins were analyzed by Western blotting with anti-His antibody to detect the amount of His_6_-tagged Osh specifically bound to MBP-tagged viral protein. (C) The split ubiquitin assay was used to test binding between p33 and yeast Osh4, 5, 6, 7 proteins in wt yeast. The bait p33 was co-expressed with the shown prey proteins. (D) Normal level of TBSV repRNA accumulation in *osh5,6,7*Δ yeast. Northern blot analysis was used to detect DI-72(+) repRNA accumulation. The accumulation level of DI-72(+) repRNA was normalized based on 18S rRNA levels. Bottom panel: Western blot analysis of the accumulation level of His_6_-tagged p33, His_6_-p92 and His_6_-Osh proteins using anti-His antibodies. See further details in Fig. 1. Each experiment was performed three times.
(EPS)

Figure S2 **Co-purification of the** ***Arabidopsis*** **Orp3A and other ORP proteins with the tombusvirus p33 replication protein from yeast cells.** Top panel: Western blot analysis of co-purified His_6_-tagged *Arabidopsis* Orp proteins expressed from plasmids with Flag-affinity purified p33 from membrane fraction of yeast. The Orp proteins were detected with anti-His antibody. The negative control was His_6_-tagged p33 purified from yeast extracts using a FLAG-affinity column. Note the presence of a faint nonspecific band, moving slightly faster than the Orp-His_6_ proteins in all samples. Middle panel: Western blot of purified Flag-p33 detected with anti-FLAG antibody. Bottom panel: Detection of His_6_-tagged cellular Orp proteins and His_6_-tagged p33 in total protein samples from yeast cells by Western blotting.
(EPS)

Figure S3 **Expression of Scs2p or its MSP/TM domains in yeast complement TBSV repRNA accumulation in** ***scs2***Δ** yeast.** (A) The split ubiquitin assay was used to test binding between p33 and Scs2p, MSP or Intermediate domains (shown schematically) in NMY51 yeast. The Scs2-derived MSP/TM construct lacks the C-terminal Δ127-218 amino acids, while Int/TM construct carries the C-terminal portion of Scs2p containing the intermediate domain and transmembrane portion, but lacks the MSP domain (Δ1-126 amino acids). The bait p33 was co-expressed with the shown prey proteins. Note that p33 can also interact with the Scs2p Int domain (intermediate portion). Therefore, p33 may interact with Scs2p without necessarily blocking the MSP region in Scs2p involved in interaction with FFAT-domain proteins like Osh proteins. (B) Northern blot analysis was used to detect DI-72(+) repRNA accumulation in yeast expressing the shown Scs2p domains. Note that pYC-HF expresses a short peptide and was used as a negative control.
(EPS)

Figure S4 **Tombusvirus replication and expression of p33, p92 and DI-72 repRNA induces MCS-like structures in the vicinity of tombusvirus-induced spherules in plant cells.** (A) Representative electron microscopic images of portions of *N. benthamiana* cells infected with CNV. Close up view of virus-induced spherules, which are marked with arrowheads, while the MCS-like structures are indicated by arrows. See further details in Fig. 7. (B) Close up view of spherules and MCS-like structures in plant cells agroinfiltrated to express p33/p92/DI-72 repRNA. The TEM images of stained ultra-thin sections show the close locations of characteristic spherules and MCS-like membranous structures.
(EPS)

Figure S5 **Detection of MT-tagged p33 replication protein in wild type yeast.** A) Electron-dense small nano-clusters associated to MT-tagged p33 reveal the presence of p33 protein molecules in globular membranous structures (black arrowheads) likely representing the sites of replication in yeast. B) In wt yeast MT-tagged p33 molecules (black arrowhead) concentrate in a vesicle-like structure that is continuous with MCS-like structure (black arrow). This image is from [15]. C) Immunogold labeling with anti-dsRNA antibodies and a 5 nm colloidal gold conjugate in combination with nano-clusters associated to MT-tagged p33 in ultra-thin sections of yeast. Note that the viral dsRNA (black arrows) are detected in association with weak globular structures formed by MT-tagged p33 molecules (sometimes seen as light gray areas and highlighted by black arrowheads), while putative MCSs with elongated MT-tagged p33 clusters running in parallel lines (black arrows) are present in the vicinity of viral dsRNAs. Bars, 50 nm.
(EPS)

Materials and Methods S1 Yeast strains and plasmids.
(DOC)
