# Supplementary material for: Co-opted Oxysterol-Binding ORP and VAP Proteins Channel Sterols to RNA Virus Replication Sites via Membrane Contact Sites
Source: PLoS Pathog. 2014 Oct 16;10(10):e1004388. doi: 10.1371/journal.ppat.1004388 (PMC4199759; doi:10.1371/journal.ppat.1004388)
Supplement: Materials and Methods S1 — Yeast strains and plasmids. (DOC) [file ppat.1004388.s006.doc]

**Supplementary material:**

**MATERIALS AND METHODS S1**

**Yeast strains and plasmids**. The yeast (*Saccharomyces cerevisiae*) strains BY4741 (*MAT*a *his3*Δ*1 leu2*Δ*0 met15*Δ*0 ura3*Δ*0*) and *scs2*∆ were obtained from Open Biosystems. Yeast strain NMY51 [*MAT*a *his3*∆ *200 trp1*-*901 leu2*-*3, 112 ade2 LYS2*::(*lexAop*)_4_-*HIS3 ura3*::(*lexAop*)_8_-*lacZ ade2*::(*lexAop*)_8_-*ADE2 GAL4*] was obtained from Dualsystems Biotech. The diploid strain InvSc1 was obtained form Invitrogen. Strains SEY6210 (*MAT*a *ura3-52 his3*∆*200 lys2-801 leu2-3,112 trp1*∆*901 suc2*∆*9*), JRY6266 (SEY6210 *osh3*∆*::LYS2 osh5*∆*::LEU2 osh6*∆*::LEU2 osh7*∆*::HIS3*), JRY6232 (SEY6210 *osh5*∆*::LEU2 osh6*∆*::LEU2 osh7*∆*::HIS3)*, JRY6207 (SEY6210 *osh6*∆*::LEU2 osh7*∆*::HIS3*) and JRY6259 (SEY6210 *osh1*∆*::URA3 osh2*Δ*::URA3 osh4*∆*::HIS3*) were obtained from Dr. Christopher T. Beh (Simon Fraser University) [1]. To generate yeast strain JRY6266 *his3*∆, the KanMX4 gene was amplified from plasmid pYM45 (Euroscarf) using primers #5489 and #5490 (Table S1). JRY6266 was transformed with the resulting PCR product and the recombinants selected on G418 plates. To generate yeast strains OSH6-6xHA and OSH7-6xHA, the 6x-HA-KanMX4 cassette was amplified from plasmid pYM14 (Euroscarf) with primers #5323/#5324 and #5326/#5327 respectively. The products were transformed into yeast strain BY4741 and the recombinant strains selected on G418 plates.

SCS2-6xHA was constructed by PCR-amplifying the 6x-HA-hphNT1 cassette from plasmid pYM16 with primers #3263/#3264 (Table S1). The resulting PCR product was transformed into BY4741 and recombinant strains were selected on hygromycin B plates. Plasmids pGBK-His33-CUP1/DI72-GAL1 and pGAD-His92-CUP1 have been described [2,3].

Plasmids pESC/DI72/His33 [4], pGAD-His92 [5] have been described before. Flag-tagged p33 was amplified from pGBKHis-Flag-p33 [3] with primers #2450 and #1403. The product was digested with *Nco*I/*Xho*I and ligated into *Nco*I/*Xho*I-digested pESC/DI72/His33 to create pESC/DI72/Flag33. To create pESC(Ura3)/DI72/His33 and pESC(Trp1)/DI72/His33, the Gal10p-DI72/Gal1p-His33 cassette was excised from pESC/DI72/His33 by *Bsp*1407/*Sac*I digestion and ligated into equally digested pESC-URA3 or pESC-TRP1 (Agilent Technologies). CIRV p36 open reading frame (ORF) was amplified with primers #900 and #3230, then digested with *Bam*HI and *Xho*I and ligated into *Bam*HI/*Xho*I-digested pESC/DI72/His33 to generate pESC/DI72/His36. Similarly, the digested PCR product was ligated into *Bam*HI/*Xho*I-digested pESC/DI72/Flag33 to generate pESC/DI72/Flag36 and into *Bam*HI/*Xho*I-digested pESC(Ura)/DI72/His33 to generate pESC(Ura)/DI72/His36. To create pGAD(Trp1)-His92-CUP1, the Cup1p-His92 cassette was excised from pGAD-His92-CUP1 [3] by *Not*I/*Xho*I digestion and ligated into similarly digested pGAD-92HFT [6]. pESC(Leu2)-Flag95-CUP1, expressing CIRV p95 protein will be described elsewhere (J. Pogany and P.D. Nagy, unpublished). pESC(Leu2)-Flag95-CUP1 was digested with *Mlu*I/*Ngo*MIV and the Cup1p-Flag-p95 cassette was ligated into *Mlu*I/*Ngo*MIV-digested pESC-TRP1 to generate pESC(Trp1)-Flag95-CUP1.

The ORFs of *OSH1*, *OSH2*, *OSH3*, *OSH4*, *OSH5*, *OSH6* and *OSH7* were PCR-amplified from yeast genomic DNA with the following primer pairs: #5121/#5122, #5123/#5124, #5125/#5126, #5127/#5128, #5129/#5130, #5131/#5132 and #5133/#5134, respectively. The PCR products were digested with *Not*I and *Nhe*I and ligated into *Not*I/*Xba*I-digested pYC2/NT-C for over-expression of His_6_-tagged Osh proteins in yeast. Additionally, the PCR products were digested with *Xma*I/*Xho*I and ligated into *Xma*I/*Xho*I-digested pGEX-His [2] for *E. coli* expression and purification.

*Arabidopsis thaliana* *ORP3A, ORP3B, ORP3C, ORP4A, ORP4B* and *ORP4C* cDNAs were PCR-amplified using primers #5477/#5478, #5427/#5428, #5583/#5584, #5424/#5425, #5577/#5578, #5580/#5581 using as template the reverse-transcription (RT) products made from *A. thaliana* total RNA with primers #5479 and #5429, respectively. The PCR products were digested with *Bam*HI and *Xho*I and ligated into *Bam*HI/*Xho*I-digested pYC2-NT-C.

The pMAL plasmids for *E. coli*-based expression of the C-terminal, soluble portion of TBSV p33 (MBP-tagged) have been described [7,8]. The ORF for RFP was PCR-amplified with primers #2691/#2663, digested with *Bgl*II and *Bam*HI and ligated into *Bam*HI-digested pYC2/NT-C. The ORFs of CNV p33 and CIRV p36 were PCR-amplified with primers #424/#1403 and #900/#3230, respectively, digested with *Bam*HI and *Xho*I and ligated into *Bam*HI/*Xho*I-digested pYC-RFP to generate pYC-RFP-p33 and pYC-RFP-p36.

The yeast *SCS2* ORF was PCR-amplified with primers #2990 and #2991, the PCR product was digested with *Bam*HI and *Xho*I and ligated into *Bam*HI/*Xho*I-digested pYC-HF to generate pYC-HF-scs2. To make the scs2-derived MSP-TM constructs (scs2Δ127-218) the MSP domain was PCR-amplified from yeast with primers #2990 / #3709 and digested with *Nhe*I. The transmembrane portion (TM) was PCR-amplified with primers #3711 / #2991 and digested with *Spe*I. Both PCR products were ligated and then PCR-amplified with primers #2990 / #2991, followed by digestion with *Bam*HI/*Xho*I and insertion into *Bam*HI/*Xho*I-digested pYC-HF. The C-terminal portion of *SCS2*, containing the intermediate domain and transmembrane portion (scs2 Δ1-126), was PCR-amplified with primers #3707 and #2991, then digested with *Bam*HI/*Xho*I and inserted into *Bam*HI/*Xho*I-digested pYC-HF. VAP genes VAP27-1 (At3g60600) and VAP27-2 (At1g08820) were PCR-amplified from *A. thaliana* total RNA by reverse-transcription (RT) with primers #3773 and #3772, respectively, using Superscript II RT (Invitrogen) followed by PCR amplification with primers #3458 / #3459 and #3456 / #3457, respectively. The obtained PCR products were digested with *Bam*HI/*Xho*I and inserted into *Bam*HI/*SalI*I-digested pPR-N-RE for split-ubiquitin yeast-two-hybrid assays, or into pYC(2p)FLAG-scs2, where *SCS2* ORF had been excised by *Bam*HI/*Xho*I digestion for expression in yeast. Additionally, the digested PCR-products were ligated into *Bam*HI/*Xho*I-digested pGD-L [9] for expression in *Nicotiana benthamiana* through agro-infiltration. Other *A. thaliana* VAP genes were similarly PCR-amplified from *A. thaliana* RNA by RT followed by PCR with the following primers: At2g23830 with #4251 (for RT) and #4249 / #4250 (for PCR), At2g45140 with #4254 (for RT) and #4252 / #4253 (for PCR), At5g47180 with #4257 (for RT) and #4255 / #4256 (for PCR), At4g00170 with #4260 and #4258 / #4259 (for PCR). The RT-PCR products were digested with *Bam*HI/*Xho*I and ligated into *Bam*HI/*SalI*I-digested pPR-N-RE.

**Supplementary figure legend:**

**Figure S1. Identification of the yeast Osh proteins interacting with the tombusvirus p33 replication protein.** (A) Co-purification of the yeast Osh3p, Osh5p and Osh6 proteins with the CNV p33 replication protein. Top panel: Western blot analysis of co-purified His_6_-tagged cellular Osh proteins with Flag-affinity purified p33 from membrane fraction. The Osh proteins were detected with anti-His antibody. The negative control was His_6_-tagged p33 purified from yeast extracts using a FLAG-affinity column. Bottom panel: Western blot of purified Flag-p33 detected with anti-FLAG antibody. Western blot of His_6_-tagged Osh1-6 proteins in the total yeast extracts using anti-His antibody. (B) Affinity binding assay to detect interaction between His_6_-tagged Osh and the MBP-tagged TBSV p33 protein (the C-terminal portion). The MBP-tagged viral protein or MBP control produced in *E. coli* was immobilized on amylose-affinity columns. Then, His_6_-tagged Osh3, 5, 6, 7 proteins expressed in *E. coli* were passed through the amylose-affinity columns with immobilized MBP-tagged proteins. The affinity-bound proteins were eluted with maltose from the columns. The eluted proteins were analyzed by Western blotting with anti-His antibody to detect the amount of His_6_-tagged Osh specifically bound to MBP-tagged viral protein. (C) The split ubiquitin assay was used to test binding between p33 and yeast Osh4, 5, 6, 7 proteins in wt yeast. The bait p33 was co-expressed with the shown prey proteins.

**Figure S2. Co-purification of the *Arabidopsis* Orp3A and Orp3B proteins with the tombusvirus p33 replication protein from yeast cells.** Top panel: Western blot analysis of co-purified His_6_-tagged cellular Orp proteins expressed from plasmids with Flag-affinity purified p33 from membrane fraction of yeast. The Orp proteins were detected with anti-His antibody. The negative control was His_6_-tagged p33 purified from yeast extracts using a FLAG-affinity column. Note the presence of a faint nonspecific band, moving slightly faster than the Orp-His_6_ proteins in all samples. Bottom panel: Western blot of purified Flag-p33 detected with anti-FLAG antibody.

**Figure S3.** Expression of Scs2p or its MSP/TM domains in yeast complement TBSV repRNA accumulation in *scs2∆* yeast. (A) The split ubiquitin assay was used to test binding between p33 and Scs2p, MSP or Intermediate domains (shown schematically) in NMY51 yeast. The bait p33 was co-expressed with the shown prey proteins. Note that p33 can also interact with the Scs2p Int domain (intermediate portion). Therefore, p33 may interact with Scs2p without necessarily blocking the MSP region in Scs2p involved in interaction with FFAT-domain proteins like Osh proteins. (B) Northern blot analysis was used to detect DI-72(+) repRNA accumulation in yeast expressing the shown Scs2p domains. Note that pYC-HF expresses a short peptide and was used as a negative control.

**Figure S4.** Tombusvirus replication and expression of p33, p92 and DI-72 repRNA induces MCS-like structures in the vicinity of tombusvirus-induced spherules in plant cells. (A) Representative electron microscopic images of portions of *N. benthamiana* cells infected with CNV. Close up view of virus-induced spherules, which are marked with arrowheads, while the MCS-like structures are indicated by arrows. See further details in Fig. 6. (B) Close up view of spherules and MCS-like structures in plant cells agroinfiltrated to express p33/p92/DI-72 repRNA. The TEM images of stained ultra-thin sections show the close locations of characteristic spherules and MCS-like membranous structures.

**Figure S5.** **Detection of MT-tagged p33 replication protein in wild type yeast**. A) Electron-dense small nano-clusters associated to MT-tagged p33 reveal the presence of p33 protein molecules in globular membranous structures (black arrowheads) likely representing the sites of replication in yeast. B) In wt yeast MT-tagged p33 molecules (black arrowhead) concentrate in a vesicle-like structure that is continuous with MCS-like structure (black arrow). This image is from (Barajas et al, *in press*). C) Immunogold labeling with anti-dsRNA antibodies and a 5 nm colloidal gold conjugate in combination with nano-clusters associated to MT-tagged p33 in ultra-thin sections of yeast. Note that the viral dsRNA (black arrows) are detected in association with weak globular structures formed by MT-tagged p33 molecules, while putative MCSs with elongated MT-tagged p33 are present in the vicinity of viral dsRNAs. Bars, 50 nm.

**Table S1. Primers used in this study.**

Name Sequence

| 424/CNV92BAM/F | CGACGGATCCGATACCATCAAGAGGATGCTGTG |
| --- | --- |
| 900/CIRV/81/F-BAM | CGACGGATCCGAGGGTTTGAAGGCTGAGTCTACCA |
| 1403/CNV/33STOP/XHO/R | GCCGCTCGAGCTATTTCACACCAAGGGACTCA |
| 2450/FLAG-UB/HINDIII/NCOI/F | GGCAAGCTTACCATGGGTCGGGATTACAAGGAC |
| 2653/SSA1\BAM\F | CGCCGGATCCTCAAAAGCTGTCGGTATTG |
| 2663/RFP MONO/BAMHI/NHEI/NOSTOP/R | GCGGATCCGCTAGCGGCGCCGGTGGAGTGGCG |
| 2691/RFPMONO/BGLII/F | CGGAGATCTATGGCCTCCTCCGAGGAC |
| 2812/SSA1/XHO/R | GGCCTCGAGTTAATCAACTTCTTCAACGGTTGG |
| 2990/SCS2/BAMHI/F | GCCGGATCCATGTCTGCTGTTGAAATTTCC |
| 2991/SCS2/NHEI/STOP/XHOI/R | CGGCTCGAGTTAGCTAGCTCTGTAGAACCATCCTAAAAC |
| 3230/CIRV/P36/XHOI/R | CCGCTCGAGCTATTTGACACCGAGGGATT |
| 3263/SCS2/CTAG/S3-HR/F | ATTGGTTGCACTCCTTATCTTGGTTTTAGGATGGTTCTACAGACGTACGCTGCAGGTCGA |
| 3264/SCS2/CTAG/S2-HR/R | ATATATATTTAGAATACAGCTATATCCTCAATCTCCCTATTAATCGATGAATTCGAGCTC |
| 3456/VAP27-2/BAMHI/F | GCCGGATCCATGAATATGCCGCTGTTGG |
| 3457/VAP27-2/XBAI/STOP/XHOI | CGGCTCGAGTCATCTAGAAGTGCGCAGAAAGTGCCCG |
| 3458/VAP27-1/BAMHI/F | GCCGGATCCATGAGTAACATCGATCTGATTGGG |
| 3459/VAP27-1/NHEI/STOP/XHOI | CGGCTCGAGTTAGCTAGCTGTCCTCTTCATAATGTATCC |
| 3707/SCS2/AA127/BAM/SPE/F | GCCGGATCCACTAGTATAAGTCCAGATGTACACC |
| 3709/SCS2/AA126/NHE/STP/XHO/R | CGGCTCGAGTTAGCTAGCCAAATATTTGACTTTTATC |
| 3711/SCS2/AA219/BAM/SPE/F | GCCGGATCCACTAGT*GAAAATGAATCATCCAGCATG* |
| 3762/ECFP/AA154/6HIS/NOSTP/BAM/R | GCCGGATCCACCATGATGATGATGATGATGAGAACCGGCGGTGATATAGACGTTG |
| 3763/ECFP/AA155/HIND/NCO/F | CGGCAAGCTTACCATGGACAAGCAGAAGAACGGC |
| 3764/ECFP/6HIS/NOSTP/BAM/R | GCCGGATCCACCATGATGATGATGATGATGAGAACCCTTATACAGCTCGTCCATGCC |
| 3772/ATVAP27-2/RT/R | GATATGATATAGACACCGA |
| 3773/ATVAP27-1/RT/R | TGAGGGATTAATTTCAAGGT |
| 3907/ECFP/HIND/NCO/6HIS/F | CGGCAAGCTTACCATGGGACATCATCATCATCATCATGTGAGCAAGGGCGAGGAGCTG |
| 4249/AT2G23830-VAP/BAMHI/F | GCCGGATCCATGAGTAACAACGAGCTTCTC |
| 4250/AT2G23830-VAP/STP/XHOI/R | CGGCTCGAGCTAACAAAAACACTGAAATATAAAC |
| 4251/AT2G23830-VAP/RT/R | CAATGAAGTAAAGAGAAAATG |
| 4252/AT2G45140-VAP/BAMHI/F | GCCGGATCCATGAGTAACGAGCTTCTCAC |
| 4253/AT2G45140-VAP/STP/XHOI/R | CGGCTCGAGTCATGTCCTCTTCATAATG |
| 4254/AT2G45140-VAP/RT/R | ATTTTGTTGAAGGGATCTG |
| 4255/AT5G47180-VAP/BAMHI/F | GCCGGATCCATGACCGGCGTTGGCGAG |
| 4256/AT5G47180-VAP/STP/XHOI/R | CGGCTCGAGTTATGTGGGAGAAGCTAAG |
| 4257/AT5G47180-VAP/RT/R | GTGTTGAAGCTCAAGAG |
| 4258/AT4G00170-VAP/BAMHI/F | GCCGGATCCATGACGACCGGAGATCTCG |
| 4259/AT4G00170-VAP/STP/XHOI/R | CGGCTCGAGTTATATCCGGTTCAATAAGTAG |
| 4260/AT4G00170-VAP/RT/R | TGAAGCTAACCCCATGGC |
| 5121/SWH1/NOT/XMA/F | GCCAGCGGCCGCCCGGGACCATGGAACAACCTGATCTATCG |
| 5122/SWH1/ST/NHE/XHO/R | CGGCTCGAGGCTAGCTTAGAAAATATCAGCACAATCTT |
| 5123/OSH2/NOT/XMA/F | GCCAGCGGCCGCCCGGGACCATGTCTAGGGAAGACTTGTC |
| 5124/OSH2/ST/NHE/XHO/R | CGGCTCGAGGCTAGCTTAAAAAATGTCACCACAATCTTTC |
| 5125/OSH3/NOT/XMA/F | GCCAGCGGCCGCCCGGGACCATGGAAACAATTGATATACAAAATC |
| 5126/OSH3/ST/NHE/XHO/R | CGGCTCGAGGCTAGCTCACCAGAGTTGAGAAATATC |
| 5127/OSH4/NOT/XMA/F | GCCAGCGGCCGCCCGGGACCATGTCTCAATACGCAAGCTC |
| 5128/OSH4/ST/NHE/XHO/R | CGGCTCGAGGCTAGCTTACAAAACAATTTCCTTTTCTTC |
| 5129/OSH5/NOT/XMA/F | GCCAGCGGCCGCCCGGGACCATGTCTCAACACGCAAGCTC |
| 5130/OSH5/ST/NHE/XHO/R | CGGCTCGAGGCTAGCTTATATAGTAATTTCGTTCTCC |
| 5131/OSH6/NOT/XMA/F | GCCAGCGGCCGCCCGGGACCATGGGCTCCAAAAAACTGAC |
| 5132/OSH6/ST/NHE/XHO/R | CGGCTCGAGGCTAGCCTATTGTTTTGCTGGGTTCTG |
| 5133/OSH7/NOT/XMA/F | GCCAGCGGCCGCCCGGGACCATGGCTCTCAATAAACTAAAG |
| 5134/OSH7/ST/NHE/XHO/R | CGGCTCGAGGCTAGCCTAATTCTTTTGGATTCCATG |
| 5323/OSH6/S3/F | AAAGAAGATGATTGAAAACGAAAAGCAGAACCCAGCAAAACAACGTACGCTGCAGGTCGA |
| 5324/OSH6/S2/R | CTAATCTATAATTTACAACAAATATCATATCCAACATATACAATCGATGAATTCGAGCTC |
| 5326/OSH7/S3/F | ATTTTCTATTCCGGCTTATAAAAAGCATGGAATCCAAAAGAATCGTACGCTGCAGGTCGA |
| 5327/OSH7/S2/R | AGTGAGAAATCGTACTAGTATAATTAAAATAGAATGAGAAGCATCGATGAATTCGAGCTC |
| 5427/ATORP3B/BAM/XMA/F | CGGCGGATCCCCCGGGATGGCTCCTAATGATCCTAAAAAAG |
| 5428/ATORP3B/SPE/STP/XHO/R | GCCGCTCGAGTTAACTAGTAGTAGACAGATCTTGGAATTG |
| 5429/ATORP3B/RT/R | CTGTCTTTTGGAGCGATACA |
| 5477/ATORP3A/BAM/XMA/F | CGGCGGATCCCCCGGGATGGCTTCTAACGATCCAAAAAAC |
| 5478/ATORP3A/SPE/STP/XHO/R | GCCGCTCGAGTTAACTAGTAGCAGAGAGATCTTGGAATTG |
| 5479/ATORP3A/RT/R | CACAACAAATGGCACCAAAG |
| 5489/HIS3/S1-HR/F | ATGACAGAGCAGAAAGCCCTAGTAAAGCGTATTACAAATGAAACGTACGCTGCAGGTCGA |
| 5490/HIS3/S2-HR/R | CTACATAAGAACACCTTTGGTGGAGGGAACATCGTTGGTACCATCGATGAATTCGAGCTC |
| 5569/OSH6/BAM/F | GCCGGATCCATGGGCTCCAAAAAACTGAC |

References:

1. Beh CT, Cool L, Phillips J, Rine J (2001) Overlapping functions of the yeast oxysterol-binding protein homologues. Genetics 157: 1117-1140.

2. Barajas D, Li Z, Nagy PD (2009) The Nedd4-type Rsp5p ubiquitin ligase inhibits tombusvirus replication by regulating degradation of the p92 replication protein and decreasing the activity of the tombusvirus replicase. J Virol 83: 11751-11764.

3. Li Z, Barajas D, Panavas T, Herbst DA, Nagy PD (2008) Cdc34p ubiquitin-conjugating enzyme is a component of the tombusvirus replicase complex and ubiquitinates p33 replication protein. J Virol 82: 6911-6926.

4. Pathak KB, Sasvari Z, Nagy PD (2008) The host Pex19p plays a role in peroxisomal localization of tombusvirus replication proteins. Virology 379: 294-305.

5. Panavas T, Nagy PD (2003) Yeast as a model host to study replication and recombination of defective interfering RNA of Tomato bushy stunt virus. Virology 314: 315-325.

6. Serva S, Nagy PD (2006) Proteomics analysis of the tombusvirus replicase: Hsp70 molecular chaperone is associated with the replicase and enhances viral RNA replication. J Virol 80: 2162-2169.

7. Rajendran KS, Nagy PD (2004) Interaction between the replicase proteins of Tomato bushy stunt virus in vitro and in vivo. Virology 326: 250-261.

8. Rajendran KS, Nagy PD (2003) Characterization of the RNA-binding domains in the replicase proteins of tomato bushy stunt virus. J Virol 77: 9244-9258.

9. Barajas D, Jiang Y, Nagy PD (2009) A unique role for the host ESCRT proteins in replication of Tomato bushy stunt virus. PLoS Pathog 5: e1000705.
